# Supplementary material for: lncRNA LOC102724169 plus cisplatin exhibit the synergistic anti-tumor effect in ovarian cancer with chronic stress
Source: Mol Ther Nucleic Acids. 2021 Mar 5;24:294–309. doi: 10.1016/j.omtn.2021.03.001 (PMC8010577; doi:10.1016/j.omtn.2021.03.001)
Supplement: Document S1. Figure S1, Table S1, and Supplemental materials and methods [file mmc1.pdf]

OMTN, Volume *24*

Supplemental information

lncRNA LOC102724169 plus cisplatin exhibit the synergistic anti-tumor effect in ovarian cancer with chronic stress

Xiaofang Zhou, Mu Liu, Guanming Deng, Le Chen, Lijuan Sun, Yun Zhang, Chenhui Luo, and Jie Tang

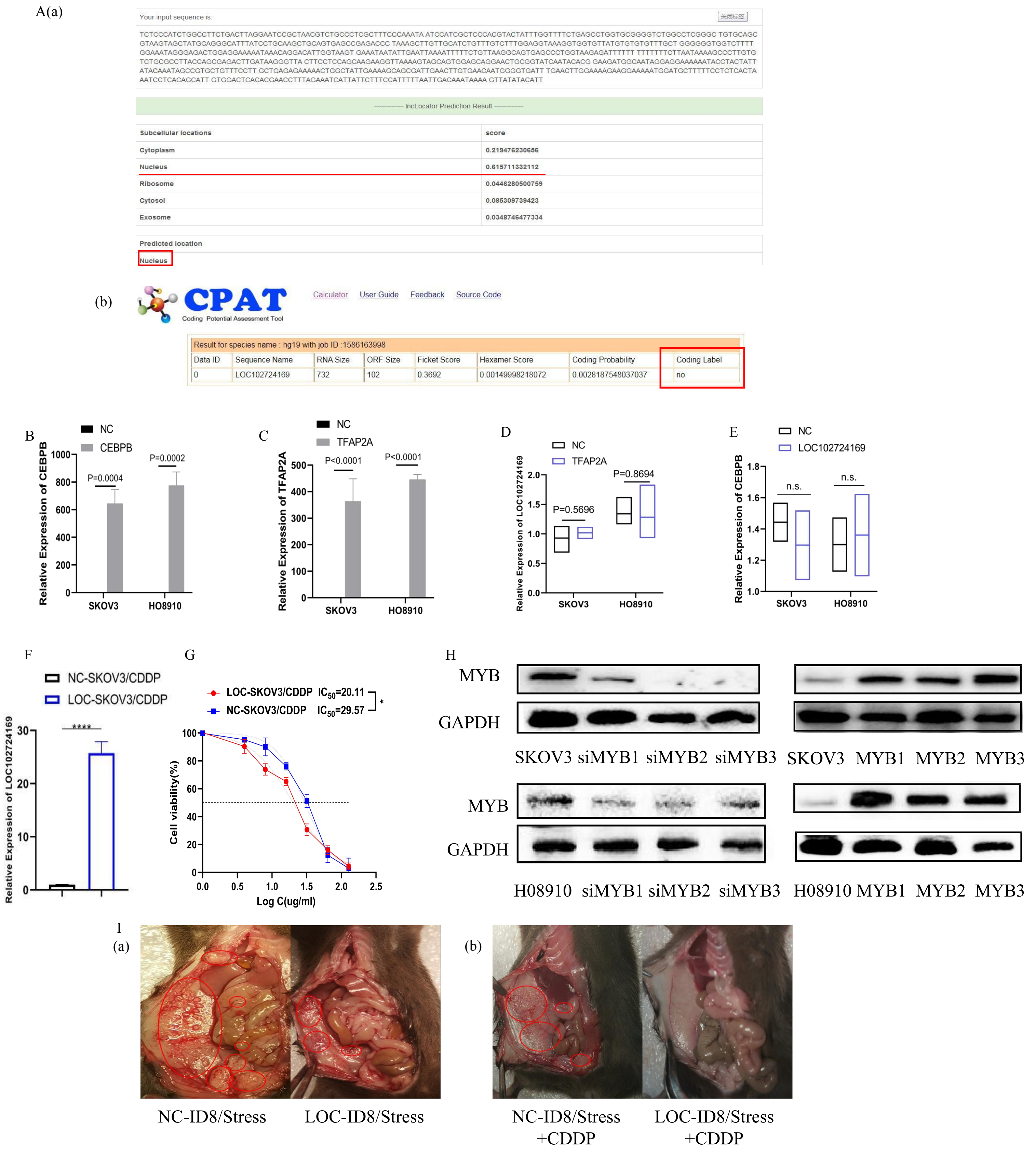

Supplementary Figure 1: A(a): lncLocator (http://www.csbio.sjtu.edu.cn/cgi-bin/lncLocator.py) suggested that LOC102724169 was mostly located in the cell nucleus. (b): A very low coding potential of LOC102724169 was predicted by CPC (coding potential calculator, http://cpc.cbi.pku.edu.cn/). B: Relative CEBPB expression in SKOV3 and HO8910 cells transfected with CEBPB expression vector or negative control, measured using RT-qPCR and normalized to GAPDH. C: Relative TFAP2A expression in SKOV3 and HO8910 cells transfected with CEBPB expression vector or negative control, measured using RT-qPCR and normalized to GAPDH. D: Relative expression of LOC102724169 in both SKOV3 and HO8910 cells transfected with CEBPB or control plasmid. E: Relative expression of CEBPB in both SKOV3 and HO8910 cells transfected with LOC102724169 or control plasmid. F: Relative expression of LOC102724169 in SKOV3/CDDP cells transfected with LOC102724169 or control plasmid. G: IC50 of SKOV3/CDDP cells transfected with LOC102724169 transcript was determined by CCK8 assays using a wide range of cisplatin concentrations. H: Relative MYB expression after SKOV3 and HO8910 cells were transfected with siRNAs targeting MYB (left panel) or MYB plasmids (right panel). I(a): NC/LOC-ID8 cells were injected intraperitoneally in stressed C57BL/6 mice respectively. Red circles marked macroscopic tumor tissue and metastases lesions. (b): LOC-ID8/stressed group and NC-ID8/stressed group treated with CDDP. Red circles marked macroscopic tumor tissue and metastases lesions.*P＜0.05, **P＜0.01. Statistical differences calculated using two-tailed student' s t-test. Data are represented as mean±SEM. CDDP: (Cisplatin).

Supplemental Table 1: PCR primer sequences

Gene official name	Forward primer	Reverse primer
GAPDH	AACGGATTTGGTCGTATTGG	TTGATTTTGGAGGGATCTCG
LOC102724169	TAAGGCAGTGAGCCCTGGTA	TCAAGTCTCGCTGGTAAGGC
HOTAIRM1	GGACGAATCGCATCCAGGAG	GCCCCTTCCTCCGCTAAATC
AKAP12	GAGATGGCTACTAAGTCAGCGG	CAGTGGGTTGTGTTAGCTCTTC
ARMC9	GAAGCTGAACGTAGGTCAGTG	CAGACGCTCTGGAGGTACTCA
LRRN4	CACACCCACCTTGCTTCAAC	CATGCAGCTATCGTGCGAGA
KCNG3	GAGTCACCTTGAGGGTACTTAGA	CATAACCATCTCTCGGTAGCAAC
FAM228B	GGCTGAAACTGCCTACAAGAT	TAGCACAGCGGTTCTCTTTCT
IGSF10	TTGGAGTTTGCCTGATGGAAC	CGCTACCCCAACTTTGTTGAAG
TGFBR3	GTGTTCCCTCCAAAGTGCAAC	AGCTCGATGATGTGTACTTCCT
CCDC170	GCTCAGTGCTGTAGAAGCAAA	TTGTGAGGTTGTGACTGCATC

mRNAs and lncRNAs reverse transcription and real-time quantitative PCR kits were purchased from Guangzhou RiboBio Biotechnology Co., Ltd.

Supplemental text 1: Information of LOC102724169

KeggID: hsa:102724169

Description: uncharacterized LOC102724169

>LOC102724169

TCTCCCATCTGGCCTTCTGACTTAGGAATCCGCTAACGTCTGCCCTCGCTTTCCCAAATA
ATCCATCGCTCCCACGTACTATTTGGTTTTCTGAGCCTGGTGCGGGGTCTGGCCTCGGGC
TGTGCAGCGTAAGTAGCTATGCAGGGCATTTATCCTGCAAGCTGCAGTGAGCCGAGACCC
TAAAGCTTGTTGCATCTGTTTGTCTTTGGAGGTAAAGGTGGTGTTATGTGTGTGTTTGCT
GGGGGGTGGTCTTTTGGAAATAGGGAGACTGGAGGAAAAATAAACAGGACATTGGTAAGT
GAAATAATATTGAATTAAAATTTTTCTGTTAAGGCAGTGAGCCCTGGTAAGAGATTTTTT
TTTTTTTCTTAATAAAAGCCCTTGTGTCTGCGCCTTACCAGCGAGACTTGATAAGGGTTA
CTTCCTCCAGCAAGAAGGTTAAAAGTAGCAGTGGAGCAGGAACTGCGGTATCAATACACG
GAAGATGGCAATAGGAGGAAAAAATACCTACTATTATACAAATAGCCGTGCTGTTTCCTT
GCTGAGAGAAAAACTGGCTATTGAAAAGCAGCGATTGAACTTGTGAACAATGGGGTGATT
TGAACTTGGAAAAGAAGGAAAAATGGATGCTTTTTCCTCTCACTAAATCCTCACAGCATT
GTGGACTCACACGAACCTTTAGAAATCATTATTCTTTCCATTTTTAATTGACAAATAAAA
GTTATATACATT
