# Supplementary material for: lncRNA LOC102724169 plus cisplatin exhibit the synergistic anti-tumor effect in ovarian cancer with chronic stress
Source: Mol Ther Nucleic Acids. 2021 Mar 5;24:294–309. doi: 10.1016/j.omtn.2021.03.001 (PMC8010577; doi:10.1016/j.omtn.2021.03.001)
Supplement: Document S2. Article plus supplemental information [file mmc2.pdf]

Molecular Therapy
Nucleic Acids
Original Article

lncRNA LOC102724169 plus cisplatin exhibit the synergistic anti-tumor effect in ovarian cancer with chronic stress

Xiaofang Zhou,[1] Mu Liu,[1] Guanming Deng,[2] Le Chen,[3] Lijuan Sun,[4] Yun Zhang,[5] Chenhui Luo,[6] and Jie Tang[1,7]

[1]Department of Gynecologic Oncology, Hunan Cancer Hospital, The Affiliated Cancer Hospital of Xiangya School of Medicine, Central South University, Changsha 410013, P.R. China; [2]Department of Gynecology and Obstetrics, Zhuhai Center for Maternal and Child Health Care, Zhuhai 519001, P.R. China; [3]Department of Gynecology and Obstetrics, Union Hospital, Tongji Medical College, Huazhong University of Science and Technology, Wuhan 430022, P.R. China; [4]Department of Gynecology and Obstetrics, Shaoyang Central Hospital, Shaoyang 422000, P.R. China; [5]Department of Pathology, Hunan Cancer Hospital, The Affiliated Cancer Hospital of Xiangya School of Medicine, Central South University, Changsha 410013, P.R. China; [6]Department of the Animal Lab, Hunan Cancer Hospital, The Affiliated Cancer Hospital of Xiangya School of Medicine, Central South University, Changsha 410013, P.R. China; [7]Hunan Gynecologic Cancer Research Center, Hunan Cancer Hospital, The Affiliated Cancer Hospital of Xiangya School of Medicine, Central South University, Changsha 410013, P.R. China

Chronic stress has been proven to accelerate the development and progression of ovarian cancer, but the underlying molecular mechanisms have not been fully elucidated. In a combination survey of ovarian cancer with chronic stress (OCCS) mouse models and high-throughput sequencing, a key lncRNA named LOC102724169 on chromosome 6q27 has been identified, which functions as a dominant tumor suppressor in OCCS. Transcriptionally regulated by CCAAT enhancer binding protein (CEBP) beta (CEBPB), LOC102724169 shows low expression and correlates with poor progression-free survival (PFS) in OCCS patients. LOC102724169 is an instructive molecular inhibitor of malignancy of ovarian cancer cells, which is necessary to improve the curative effect of cisplatin therapy on ovarian cancer. This function stems from the inactivation of molecules in phosphatidylinositol 3-kinase (PI3K)/AKT signaling, repressing MYB expression and retaining the responsiveness of cancer cells to cisplatin. These findings provide a mechanistic understanding of the synergistic anti-tumor purpose of LOC102724169 as a bona fide tumor suppressor, enhancing the therapeutic effect of cisplatin. The new regulatory model of "lncRNA-MYB" provides new perspectives for LOC102724169 as a chronic stress-related molecule and also provides mechanistic insight into exploring the cancer-promoting mechanism of MYB in OCCS, which may be a promising therapeutic strategy for ovarian cancer.

INTRODUCTION

Epithelial ovarian cancer (EOC) is one of the most lethal gynecological cancers globally. The reason for the high mortality of ovarian cancer is mainly due to its high rate of resistance to cisplatin and the advanced stage of disease at diagnosis.[1,2] Animal studies and other research have indicated that chronic stress leads to greater tumor burden and enhances the invasive growth ability of EOC cells, thereby resulting in a worse prognosis.[3–5] Chronic stress has long been speculated to change the gene expression of diseases[6] and even influence the progression of cancer.[7] Laboratory animal models of chronic stress have provided significant insights into changes in the neuroendocrine system that can modulate the functional activities and malignant features of tumor cells.[8] In mice models of ovarian carcinoma, the stress-induced release of catecholamines can activate the neuroendocrine system, leading to increased expression of VEGF, interleukin (IL)-6, and matrix metalloproteinase, which promote angiogenesis processes and more aggressive growth of EOC cells.[3,9] Based on above the evidence, beta blockers are offered as clinical therapy in ovarian cancer patients and prolong their overall survival[10,11] as well as disease-specific survival.[11] Better prognosis has also been observed in breast cancer and malignant melanoma using them.[12,13] However, other studies show that taking beta blockers does not significantly prolong survival in ovarian cancer patients.[14–16] This suggests that some other major underlying mechanisms are still unclear in ovarian cancer with chronic stress (OCCS).

Long non-coding RNAs (lncRNAs) function in various biological processes[17] and are also crucial players in tumor development through several not fully characterized mechanisms.[18] Recent studies have reported that lncRNAs can act as oncogenes or tumor suppressors, and their aberrant expressions are closely linked with cell migration, angiogenesis, and drug resistance in ovarian cancer.[18,19] H19 is a metastatic lncRNA that is correlated with the recurrence of high-grade serous ovarian cancer and is implicated in acquired cisplatin resistance.[20] MALAT1 is also available to promote cell proliferation and metastasis via the phosphatidylinositol 3-kinase (PI3)/AKT pathway in EOC.[21] However, as a tumor

Received 6 November 2020; accepted 2 March 2021;
https://doi.org/10.1016/j.omtn.2021.03.001.

Correspondence: Jie Tang, MD, PhD, Department of Gynecologic Oncology, Hunan Gynecologic Cancer Research Center, Hunan Cancer Hospital, The Affiliated Cancer Hospital of Xiangya School of Medicine, Central South University, 283 Tongzipo Road, Yuelu District, Changsha 410013, P.R. China.
E-mail: tangjie@hnca.org.cn

 © 2021 The Author(s).
This is an open access article under the CC BY license (http://creativecommons.org/licenses/by/4.0/).

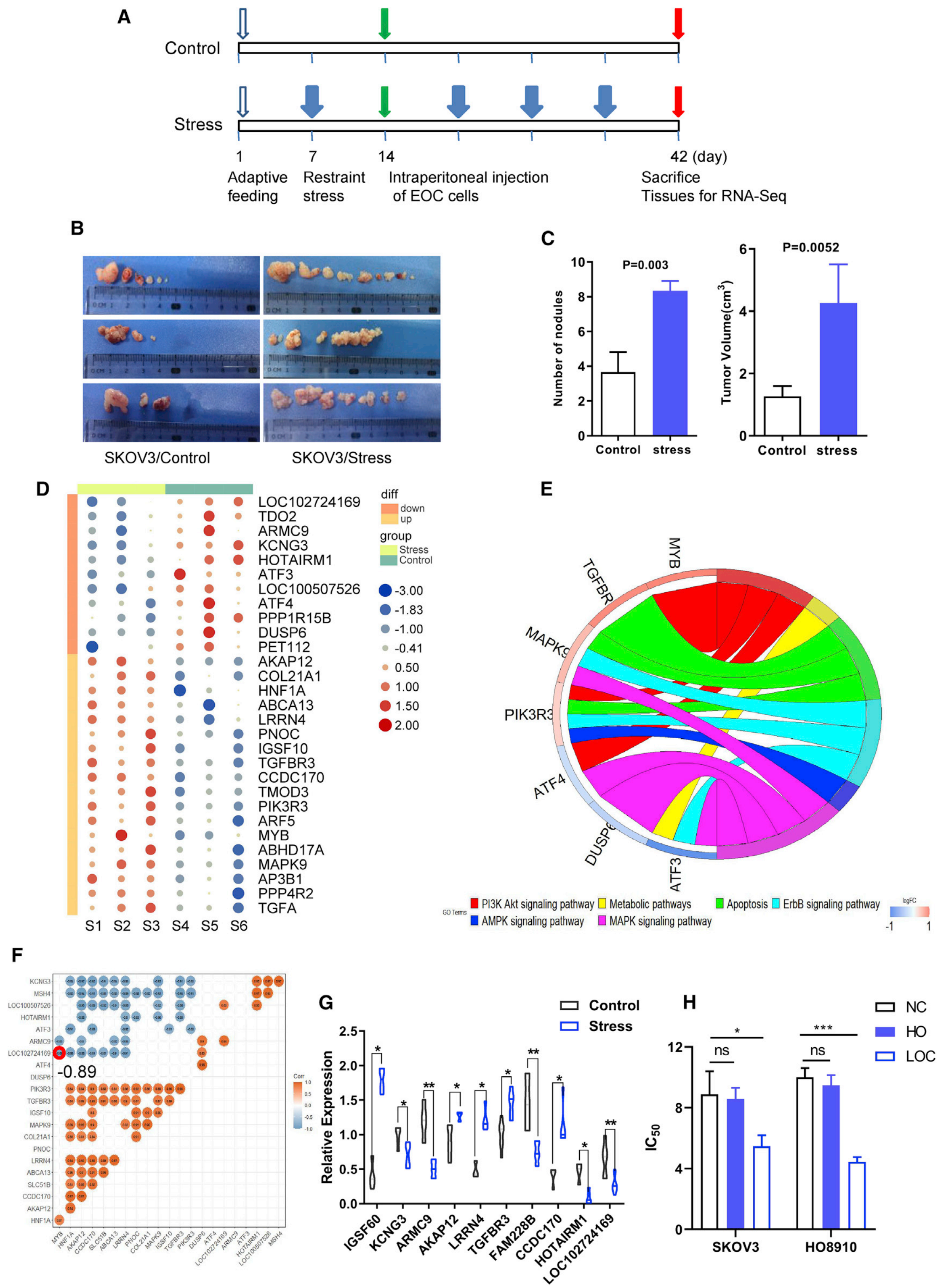

Figure 1. Chronic stress-related lncRNAs identified through integrated analysis in ovarian cancer

(A) Schematic diagram of stress nude mouse model. Blue open arrows indicate that mice were randomly divided into the stress group or the control group after adaptive growth for 1 week. Blue filled arrows indicate the stressed group, in which mice receive 2 h of immobilization daily. Green arrows indicate that SKOV3 cells were injected intraperitoneally in nude mice with/without periodic immobilization. Red arrows indicate mice were euthanized, and tumor tissues were collected for RNA sequencing. (B)

(legend continued on next page)

suppressor, lncRNA GAS5 is shown to have low expression in EOC tissues and inhibit tumor metastasis through the GAS5-E2F4-PARP1-mitogen-activated protein kinase (MAPK) axis.[22] HOXA11-AS is also lowly expressed in EOC tumor tissues and exerts a tumor suppressor function, which can be improved by the T allele.[23] Clearly, lncRNAs have a prominent role in ovarian cancer, which leads us to postulate whether lncRNAs can regulate the progression of OCCS.

In this study, we established a mouse model of chronic stress that faithfully replicates the development and progression of ovarian cancer suffering chronic stress and isolated deregulated lncRNAs involved in OCCS. A new and dominant lncRNA tumor suppressor named LOC102724169 was identified. We explored whether and how LOC102724169 might negatively regulate the aggressiveness of OCCS, and how such mechanisms might enhance the synergistic anti-tumor effect to improve cisplatin response in the context of OCCS. To our knowledge, this is the first time that any study has illustrated the roles of lncRNAs in OCCS, thereby increasing knowledge of lncRNA-related regulatory mechanisms of OCCS and providing a new approach for the clinical treatment of ovarian cancer.

RESULTS

Chronic stress-related lncRNAs identified through integrated analysis in ovarian cancer

We stressed female nude mice with periodic immobilization[3] for a week before SKOV3 cells were injected intraperitoneally in all nude mice. The stress effects on tumor growth and metastasis were examined first (Figures 1A and 1B). In mice receiving 2 h of immobilization daily, the number of tumor nodules doubled compared to those in the control group (Figure 1C), while the mean tumor volume increased more than 2-fold (Figure 1C). As expected, restraint stress has significantly accelerated the progression of ovarian cancer. Given that lncRNAs play vital roles during the process of ovarian cancer, we performed transcriptome profiling on tumor tissues from stressed mice and a matched control group using high-throughput sequencing to more comprehensively survey whether lncRNAs are involved in OCCS. We observed a total of 181 mRNAs and lncRNAs to be differentially expressed, some of which are shown in Figure 1D. These differentially expressed genes were enriched in Kyoto Encyclopedia of Genes and Genomes (KEGG) pathways such as apoptosis, ABC transporters, the phosphatidylinositol 3-kinase (PI3K)/AKT pathway, and the MAPK pathway, which are linked to either multi-drug resistance or oncogenesis (Figure 1E). Of note, several differentially expressed genes related to drug resistance were identified, including ABC transporters (*ABCA13*), P-type ATPase (*ATP11B*), and protein phosphatase family genes. Additionally, a clustering algorithm was used to calculate the correlation between genes (Figure 1F). We randomly validated several genes such as *IGSF10*, *TGFBR3*, and others (Table S1) from 181 mRNAs and lncRNAs mentioned above in the other mouse tumor tissues (Figure 1G), and we found that the result was consistent with what we observed in the high-throughput sequencing (Figure 1D). These findings indicated that the mouse model was built on meaningful data in the context of OCCS. Since KEGG pathway analysis is mainly concentrated on cell apoptosis and drug resistance, we tried to explore a key lncRNA with clinical therapeutic significance by first detecting cell viability. Half-maximal inhibitory concentration (IC_{50}) was determined by a Cell Counting Kit-8 (CCK-8) using a wide range of cisplatin concentrations. We tested all five different lncRNAs and found that only LOC102724169 reduced the value of IC_{50} in ovarian cancer cells (Figure 1H), indicating that the inferred lncRNA might have a vital role in OCCS.

Identification of lncRNA LOC102724169 transcriptionally regulated by CEBPB

We chose lncRNA LOC102724169, an uncharacterized new lncRNA, for further study. LOC102724169 is a 10-kb gene located between PRR18 (NCBI: NC_000006.12 [166,305,300–166,308,392]) and LOC100289495 (NCBI: NC_000006.12 [166,342,631–124166,351, 469]) on human chromosome 6q27 (Figure 2A). It is composed of two exons with a transcript length of 732 nt (Supplemental materials and methods). Furthermore, lncLocator (http://www.csbio.sjtu.edu.cn/bioinf/lncLocator/) suggested that LOC102724169 was mostly located in the cell nucleus (Figure S1A), and RNA *in situ* hybridization (ISH) was then conducted to validate the location of LOC102724169 in SKOV3 cells (Figure 2B). As a non-coding RNA, LOC102724169 has no open reading frames (ORFs) larger than 200 bp.[24] A very low coding potential of LOC102724169 was predicted, and the CPC (coding potential calculator, http://cpc2.gao-lab.org)[25] computational algorithm predicted that LOC102724169 has a very low coding potential (Figure S1A), similar to GAS5, a well-known lncRNA. However, there is no information about the function of LOC102724169.

We sought to identify the molecular mechanism that resulted in the low expression of LOC102724169 in tumor tissues of OCCS. JASPAR (http://jaspar.genereg.net/) was used to predict potential transcription factors that could bind to the promoter regions of LOC102724169. Although there are many transcription factor binding sites in LOC102724169 promoters, we selected the highest score and the highest relative score genes, TFAP2A and CCAAT enhancer binding protein (CEBP) beta (CEBPB), for further verification

Gross specimens of tumor tissue in stressed mice and control group. (C) Quantification of tumor nodules and tumor volume in stressed mice and the matched control group. (D) Heatmap shows part of differentially expressed mRNAs and lncRNAs in high-throughput sequencing of stressed mice and the matched control group. (E) Part of the KEGG pathway and their enriched genes of high-throughput sequencing in stressed mice and control group. (F) Correlation analysis of differentially expressed genes in sequencing results. (G) Several mRNAs and lncRNAs from high-throughput sequencing were validated in mice tumor tissue between the stress and control groups. (H) Cell viability of LOC102724169 (LOC) and HOTAIRM1 (HO) compared to the negative control (NC) detected by CCK-8. LOC102724169 reduced the value of IC_{50} in both SKOV3 and HO8910 cells. *p < 0.05, **p < 0.01, ***p < 0.001. Statistical differences were calculated using a two-tailed Student's t test. Data are represented as mean ± SEM.

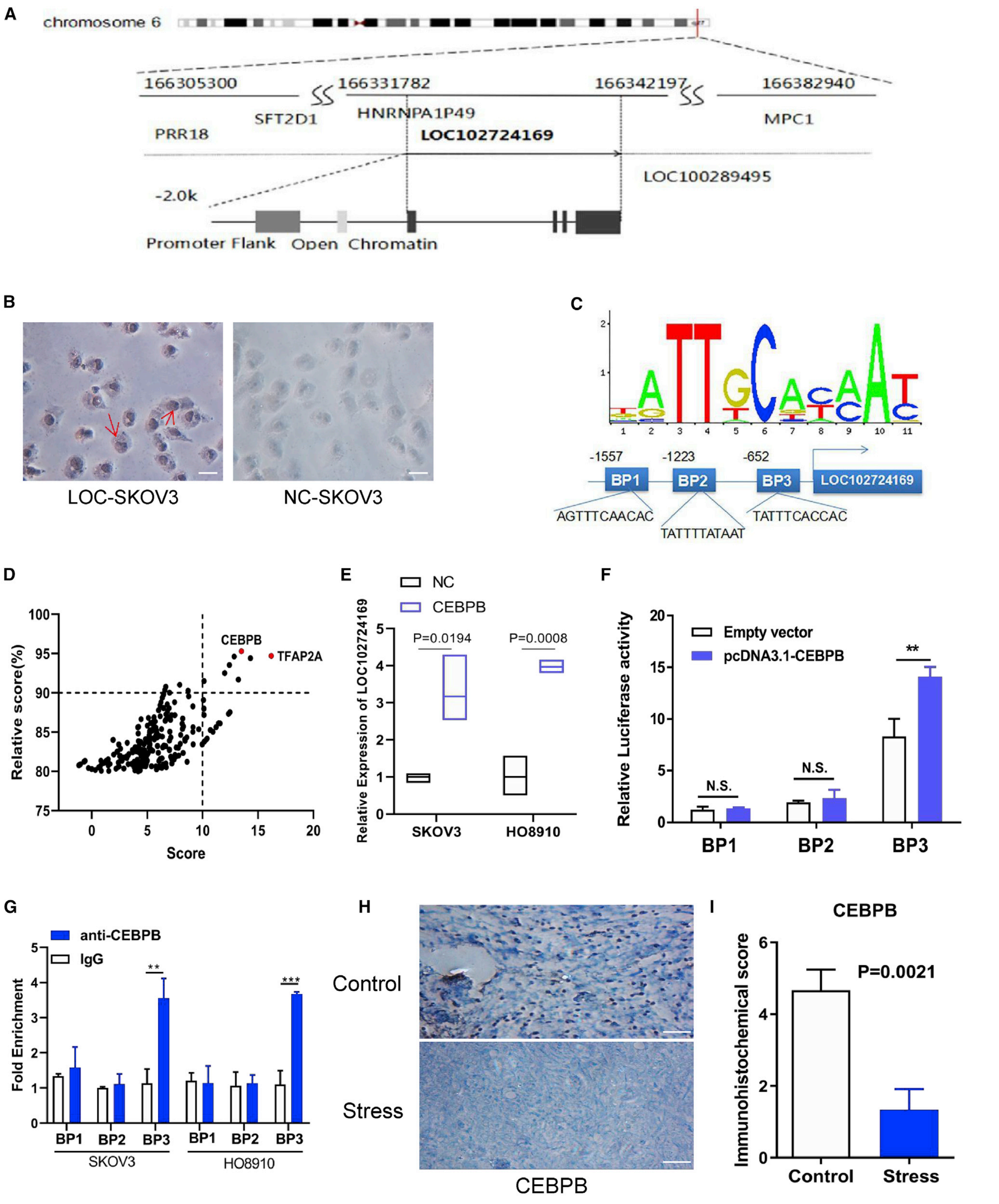

(legend on next page)

(Figure 2D). In the preliminary validation of qRT-PCR, only CEBPB's binding capacity improved the LOC102724169 expression (Figures 2E and S1B–S1D). There were several CEBPB binding sites in the promoter of LOC102724169 predicted by JASPAR. We focused on three CEBPB binding sites (BP1 [−1567 to −1557], 5′-AGTTTCAACAC-3′; BP2 [−1233 to −1223], 5′-TATTTTATAAT-3′; BP3 [−662 to −652], 5′-TATTTCACCAC-3′) with the highest scores (Figure 2C). LOC102724169 expression increased in EOC cells after being transfected with CEBPB plasmid as compared with the control cells (Figure 2E), while overexpressed LOC102724169 did not significantly increase CEBPB expression (Figure S1E); this implied that CEBPB regulated LOC102724169 in a unidirectional way. Next, to validate the exact binding region of CEBPB, we constructed three luciferase report plasmids that contained three regions respectively. These plasmids were transfected into SKOV3 cells separately, and the results indicated that there was a marked CEBPB binding activity in the BP3 site (5′-TATTTCACCAC-3′) of LOC102724169 (Figure 2F). The specific association between LOC102724169 and CEBPB was further validated by chromatin immunoprecipitation (ChIP) and qRT-PCR in EOC cells (Figure 2G). To investigate whether CEBPB was also aberrantly expressed in OCCS, CEBPB expression was measured in tumor tissues of two groups by immunohistochemistry (IHC) analysis (Figure 2H). The results indicated that CEBPB expression was significantly lower in stressed mice tumor tissues compared with non-stressed mice tumor tissues (Figure 2I). These experiments demonstrated that LOC102724169 expression was positively regulated by CEBPB in OCCS.

Chronic stress promoted tumor progression in EOC patients

We examined CEBPB and LOC102724169 expression among human EOC tumor tissues and benign ovarian tumor (BOT) tissues and found that both had lower expression in EOC patients (Figures 3A and 3B). Consistent with our observations, LOC102724169 was positively correlated with CEBPB (Figure 3C). To note the difference in OCCS patients, assessment of depressive symptoms caused by chronic stress was conducted 1–7 days before tumor resection among 71 EOC patients. Using established threshold values of a nine-item depression scale from the Patient Health Questionnaire (PHQ-9) ≥ 5,[26,27] we identified that 28 participants (39%) had high-risk factors related to chronic stress. Because chronic stress can worsen ovarian cancer,[5,28] we reasoned that chronic stress would be associated with clinical and pathological features of EOC patients. As shown in Table 1, high-risk patients have more invasive EOC characteristics, such as International Federation of Gynecology and Obstetrics (FIGO) stage, more residual disease, and cisplatin resistance ($p < 0.05$). Correlation analysis revealed that chronic stress was positively associated with cisplatin resistance (Figure 3E). To further investigate the clinical significance of chronic-stress-related LOC102724169, we assessed LOC102724169 expression in tumor tissues from EOC patients between those two groups and found significantly lower expression of LOC102724169 in the high-risk group (Figure 3D). We also explored the relationships of stress status, clinical-pathological parameters, and postoperative residual disease with LOC102724169 expression in EOC patients. Lower expression of LOC102724169 was seen in the high-risk stress group and was associated with more residual disease ($p < 0.05$) in Table 2. Additionally, we observed that a low LOC102724169 level correlated significantly with poorer progression-free survival (PFS) of EOC patients through Kaplan-Meier analysis (Figure 3F). Univariate Cox regression analysis showed that poor prognosis was observed in EOC patients with high-grade tumor, FIGO stage III/IV, lymph node metastasis, residual disease, chronic stress, cisplatin resistance, and low LOC102724169 expression. Additionally, a multivariate Cox regression analysis suggested that these factors, especially FIGO stage III/IV, chronic stress, cisplatin resistance, and low LOC102724169 expression, were independent risk predictors of prognosis for EOC patients (Table 3). Collectively, these data showed that LOC102724169 might exert a critical influence in OCCS patients.

LOC102724169 was necessary for reducing cell viability and inducing apoptosis of EOC cells

To determine whether LOC102724169 can play a pivotal functional role in OCCS, we first detected the ontology expression of LOC102724169 between ovarian cancer cell lines and normal ovarian epithelial cells. LOC102724169 expression was much lower in SKOV3 and HO8910 (Figure 4A). We then constructed an ectopic expression vector containing the full-length LOC102724169 transcript in EOC cells (Figure 4B) and examined the effect of LOC102724169 on cellular biological functions.

Based on our sequencing results, the OCCS mice model displayed a significantly dysregulated PI3K/AKT pathway, apoptosis, and ABC transporter signaling (Figure 1E), which were linked to drug resistance. We theorized that LOC102724169 might affect the sensitivity of ovarian cancer cells to cisplatin in OCCS. Cell viability was detected, and we observed that LOC102724169 improved the response rates of ovarian cancer cells to cisplatin, exhibiting a prominent decrease of IC_{50} compared with control cells (Figures 4C, 4D, S1F,

Figure 2. Identification of lncRNA LOC102724169 transcriptionally regulated by CEBPB

(A) Gene map of LOC102724169, a lncRNA on human chromosome 6q27. (B) RNA ISH assay suggested that LOC102724169 was mostly located in the nucleus of SKOV3 cells. Original magnification, ×200; scale bars, 5 μm). (C) JASPAR (http://jaspar.genereg.net/) was used to predict sites of putative CEBPB binding motif in the LOC102724169 promoter region. (D) Relative score and score of transcription factors predicted by JASPAR. (E) Relative expression of LOC102724169 in both SKOV3 and HO8910 cells transfected with CEBPB or control plasmid. (F) Dual-luciferase reporter assays examining the relative luciferase activities, which show a marked CEBPB binding activity in the BP3 site of LOC102724169. (G) ChIP assays of the enrichment of CEBPB on the LOC102724169 promoter relative to control IgG in both SKOV3 and HO8910 cells. (H) CEBPB expression levels measured in tumor tissues of stressed mice and non-stressed mice by immunohistochemistry analysis. Original magnification, ×200; scale bars, 5 μm). (I) Immunohistochemical score of CEBPB in stressed mice and matched control group. $^{**}p < 0.01$. Statistical differences were calculated using a two-tailed Student's t test. Data are represented as mean ± SEM. ISH, *in situ* hybridization; BP3, binding position 3.

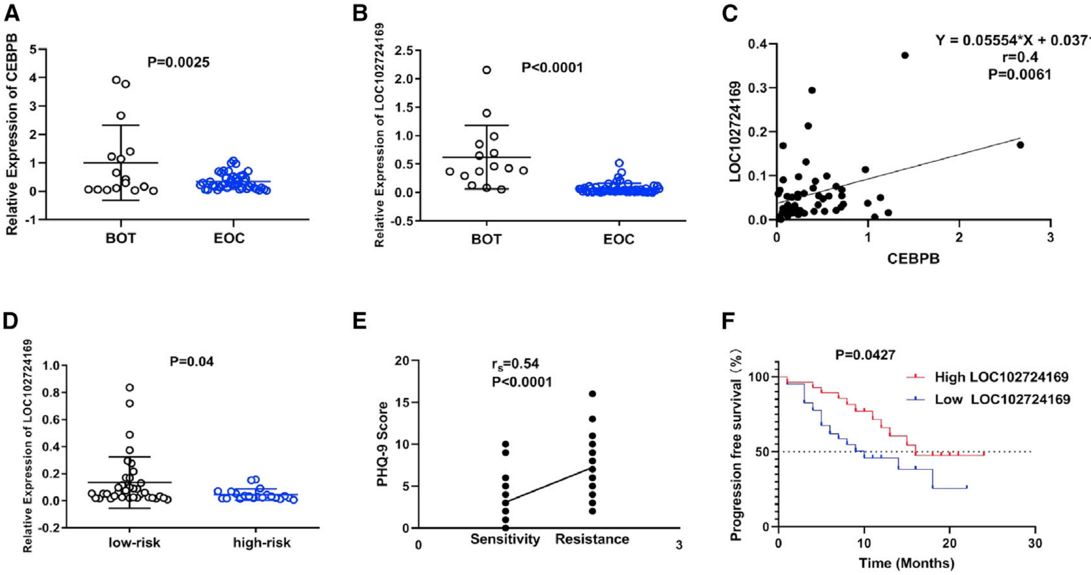

Figure 3. Chronic stress promoted tumor progression in EOC patients

(A) Clinical sample validation of CEBPB expression in BOT and EOC. (B) Clinical sample validation of LOC102724169 expression in BOT and EOC. (C) Correlation analysis of CEBPB and LOC102724169 expression levels by a Pearson correlation coefficient ($r = 0.4$, $p = 0.0061$). (D) Validation of LOC102724169 expression between high-risk stress group and low-risk stress group. LOC102724169 showed low expression in the high-risk group. (E) Spearman rank regression was used to analyze the correlation between chronic stress and cisplatin resistance. (F) Kaplan-Meier analysis and a log-rank test were used to show progression-free survival curves of EOC patients with different expressions of LOC102724169. Data are represented as mean ± SEM. BOT, benign ovarian tumor; EOC, epithelial ovarian cancer.

and S1G), which was consistent with clinical results (Figure 3D). Meanwhile, we found that overexpressed LOC102724169 alone or plus cisplatin affected the level of apoptosis markers (caspase-3) compared to control groups using caspase-3 activity assay (Figures 4E and 4F) and western blot (Figure 5G). Taken in aggregate, the dominant effect of LOC102724169 during the OCCS process was to induce apoptosis and then reduce cancer cell viability, thus exerting an extra anti-tumor effect in conjunction with cisplatin.

LOC102724169 regulated the expression of MYB and affected the PI3K/AKT pathway in OCCS

To verify the key molecules that mediate the function of LOC102724169 in OCCS, we used a strategy looking for the overlap of three independent datasets, which included two expression profiles of cisplatin resistance in ovarian cancer (Gene Expression Omnibus [GEO]: GSE33482 and GSE58470),[29] along with our high-throughput sequencing. MYB was not only the remarkably upregulated gene in the two GEO datasets but also the one overexpressed in the stressed group of high-throughput sequencing. Moreover, correlation analysis of our high-throughput sequencing showed that MYB was negatively correlated with LOC102724169 (Figure 1F, $r = -0.89$, $p < 0.05$). We thus hypothesized that MYB might be the target of LOC102724169 (Figure 5A). To confirm the relationship between LOC102724169 and MYB in OCCS, we first demonstrated that overexpression of LOC102724169 markedly downregulated the expression of MYB in EOC cells (Figures 5B and 5G), while overexpressed MYB had no significant effect on LOC102724169 expression (Figure 5C). MYB was consistently overexpressed in EOC patients with high-stress risk (Figure 5E), while it was negatively correlated with LOC102724169 expression (Figure 5F).

Given that MYB participated in multiple processes of tumorigenesis,[30] such as cell growth, angiogenesis, and resistance to apoptosis,[31,32] we asked whether LOC102724169 could exert its collaboration effect on enhancing the sensitivity of cisplatin in EOC cells by affecting the expression of MYB. The effects of MYB on the sensitivity of EOC cells to cisplatin were detected. We noticed a concurrent decrease in cell viability when EOC cells were transfected with siRNAs targeting MYB (Figures 5D and S1H). Furthermore, reinforced MYB expression in EOC cells with LOC102724169 overexpression enhanced their viability compared with controls (Figure 5D). Altogether, our data demonstrated that MYB was a clear target of LOC102724169 in increasing the response rates of EOC cells to cisplatin.

To further understand the molecular mechanisms underlying the anti-tumor effect of LOC102724169 in modulating MYB, we found that the PI3K/AKT pathway was the most enriched pathway through KEGG pathway enrichment analysis of high-throughput sequences; MYB was dysregulated in this pathway (Figure 1D). We then assumed that LOC102724169 might inhibit MYB by inactivation of PI3K/AKT signaling. As shown in Figure 5G, PI3K and the phosphorylation AKT levels were diminished markedly by LOC102724169 overexpression compared to the control group. Moreover, treatment with cisplatin further reduced their expressions in both NC-SKOV3 and LOC-SKOV3 cells (Figure 5G). A similar pattern of their expression

Table 1. Relationship between the status of chronic stress and clinical-pathological characteristics of EOC patients

Characteristics	No. of cases	Status of chronic stress: Non-stress (n = 43)	Stress (n = 28)	p value
Age (years)				
<55	39	23	16	0.762
≥55	32	20	12	
FIGO stage				
I–II	17	16	1	0.001**
III–IV	54	27	27	
Grade				
G1/G2	15	10	5	0.586
G3	56	33	23	
Lymph node metastasis				
Negative	33	22	11	0.327
Positive	38	21	17	
Residual disease				
R0	43	30	13	0.049*
Non-R0	28	13	15	
Cisplatin effects				
Sensitivity	51	42	9	0.000****
Resistance	20	4	16	
Expression of LOC102724169				
Low expression	41	20	21	0.018*
High expression	30	23	7	

EOC, epithelial ovarian cancer; R0, residual disease <0 cm.*$p < 0.05$, **$p < 0.01$, ***$p < 0.001$, ****$p < 0.0001$.

was detected in those different groups of HO8910 cells. Additionally, to further explore the involvement of the PI3K/AKT signaling, we treated EOC cells with LY294002, a highly selective PI3K inhibitor. LY294002 dramatically decreased the phosphorylation level of AKT (Ser473) in EOC cells with or without LOC102724169 overexpression. MYB was also remarkably decreased by LY294002 treatment (Figure 5G). These data collectively indicate that LOC102724169 inhibited MYB expression in OCCS by attenuating PI3K/AKT signaling.

LOC102724169 overexpression enhanced the effect of cisplatin on EOC *in vivo*

Finally, we returned to verify the roles of LOC102724169 in the OCCS mouse model (Figure 6A). After stressing and injecting SKOV3 cells intraperitoneally to induce tumor formation, we injected LOC102724169-loaded vectors (LOC-virs) or empty vector viruses (negative control [NC]-virs) through the tail vein in the OCCS nude mouse model. It was found that LOC-virs significantly diminished the tumor burden compared to the negative control (Figures 6B and 6C). To study the synergistic anti-tumor effect of LOC102724169 combined with cisplatin in OCCS, we treated both the LOC-virs group and the NC-virs group with cisplatin simultaneously. These mouse models revealed that the group with LOC102724169 overexpression was strikingly more sensitive to cisplatin therapy. Also, fewer numbers of tumor nodules and distant metastases were noted in the LOC-virs plus cisplatin group compared with the control group (Figure 6D). From the statistics, we found that the average tumor volume in the LOC-virs group was 62% less than that in the NC-virs group (Figure 6E), and it was reduced by 81% in LOC-virs with cisplatin treatment group compared to the NC-virs with the cisplatin treatment group (Figure 6E). Significant differences were also found in tumor weight among those groups (Figure 6F). Concurrently, we detected a stark difference in MYB, PI3K, and the phosphorylation AKT level among those groups by western blot (Figure 6H). Additionally, the expression of LOC102724169 was also repeated to confirm the effect in NC-virs, LOC-virs, and those plus cisplatin groups (Figure 6B).

We also used ID8 cells overexpressed with LOC102724169 (LOC-ID8) or empty vector (NC-ID8) and C57BL/6 mice with complete immune systems to validate the anti-tumor efficacy of LOC102724169. As expected, similar situations in nude mice occurred in the NC-ID8 C57BL/6 mice with bloody ascites and extensive abdominal wall metastasis, but less in either group treated with LOC102724169 or cisplatin alone, and least in mice treated with LOC102724169 plus cisplatin (Figures 6I–6K and S1I). These indicated that LOC102724169 did increase the response rate to cisplatin and exert its synergic anti-tumor efficacy *in vivo*.

DISCUSSION

Chronic stress has been known to promote the development and progression of multiple cancers, such as ovarian,[3–5] breast,[33] hepatocellular,[34] and even oral cancer.[35] Most of these studies have demonstrated its promotive effects on tumor growth, angiogenesis, and metastasis through the immune and neuroendocrine systems.[3,8,33–37] Although previous research on chronic stress worsening ovarian cancer has been shown in various species, including mice and humans,[3–5] the involved mechanistic understandings are not completely illustrated within clinical parameters; for instance, suboptimal cytoreductive surgery and chemotherapy resistance. So far, despite the exploration of the hypothalamic-pituitary-adrenal (HPA) axis, there are few studies about the functional role and mechanism of chemotherapy resistance linked to OCCS. In clinical observations, we were surprised to find that ovarian cancer patients at high risk of chronic stress are more likely to relapse with cisplatin (CDDP) resistance ($r_s = 0.54$, $p < 0.0001$, Figure 3E). Meanwhile, we established a classical restraint stress mouse model to mimic human OCCS and used tumor tissue for high-throughput sequencing. Through KEGG pathway analysis, a series of genes were screened to be enriched in apoptosis and drug resistance-related signaling pathways. Taking these interesting findings into consideration, we postulated that chronic stress might aggravate the process of ovarian cancer by reducing its response to cisplatin. Next, we used both nude mice and C57BL/6 mice to construct restraint stress mouse models treated with cisplatin for initial testing.

Table 2. Relationship between LOC102724169 expression and clinical-pathological characteristics of EOC patients

Characteristics	No. of cases	Expression of LOC102724169		p value
		Low (n = 41)	High (n = 30)	
Age (years)				
<55	39	23	16	0.817
≥55	32	18	14	
FIGO stage				
I–II	17	8	9	0.306
III–IV	54	33	21	
Grade				
G1/G2	15	6	9	0.117
G3	56	35	21	
Status of chronic stress				
Non-stress	43	20	23	0.018*
Stress	28	21	7	
Lymph node metastasis				
Negative	33	20	13	0.649
Positive	38	21	17	
Residual disease				
R0	43	20	23	0.018*
Non-R0	28	21	7	

*$p < 0.05$.

Similar tumor status was observed in both the NC-SKOV3/NC-SKOV3+cisplatin and NC-ID8/NC-ID8+cisplatin groups of our stressed mice models, in which the therapeutic efficacy of cisplatin was nearly negligible (Figures 6B and S1G). To our knowledge, this is the first time we observed that chronic stress might induce cisplatin resistance in ovarian cancer patients. At present, researchers mainly use secondary drug-resistant models through transplanting drug-resistant cell lines into animals. Furthermore, there is a lack of animal models that can better reflect the primary drug resistance for ovarian cancer. At present, we are collecting and establishing types of chronic stress mouse models, such as chronic variable stress, social isolation, and behavioral despair, to verify the clinical phenomenon we have observed above.

lncRNAs can act as tumor suppressors or oncogenes.[38,39] However, how lncRNAs may relate to the connection between stress, ovarian cancer, and chemotherapy is unknown. The present study identified how lncRNA LOC102724169 is mediated in OCCS, which expanded our understanding of the mechanisms by which chronic stress could regulate cancer pathogenesis. In our study, we showed that stress response lncRNAs could also affect the apoptosis and drug resistance of ovarian cancer. Furthermore, the dominant lncRNA LOC102724169 on chromosome 6q27, with tumor suppressor characteristics in OCCS, was identified from its location; it had no protein-coding ability, which was predicted by the CPC as another lncRNA discovery.[24,25] Moreover, we screened CEBPB as a regulator of LOC102724169 expression. JASPAR marked a very high score in CEBPB among the potential transcription factors, which suggested the high possibility of CEBPB's involvement in the regulation of LOC102724169 expression. A dual-luciferase reporter assay and ChIP further confirmed this prediction (Figures 2F and 2G). CEBPB is a member of the CEBP family of transcription factors, which has been reported to regulate cell proliferation, metastasis, and apoptosis in various types of cancer.[40–42] Epigenetically, CEBPB expression is significantly lower in stressed tumor tissues compared with the control group (Figure 2H), and overexpressing CEBPB caused increases in LOC102724169 expression in EOC cells (Figure 2D). These data suggest that CEBPB can exert a promoting effect on LOC102724169 expression in EOC cells. Overall, our results pointed to the applicability of LOC102724169 as a tumor suppressor in OCCS, where CEBPB is a transcription factor during ontogeny, and which may result in the low expression of LOC102724169.

In recent years, bevacizumab has been shown to effectively block the interaction of VEGF with its receptor, with response rates ranging from 16% to 18% in both platinum-sensitive and platinum-refractory EOC patients.[43] However, it was found that the efficacy rate of EOC patients was still no more than 30% even when bevacizumab and cisplatin were combined.[44] Researchers have studied the role of lncRNAs in the chemoresistance of various cancers.[45,46] Our study explored the role of LOC102724169 combined with cisplatin in OCCS. Based on the bioinformatics analysis, we found that MYB might be involved in the response to cisplatin treatment of EOC cells.

MYB functions as a proto-oncogene in multiple types of cancers.[30] For example, c-Myb is important for the continued proliferation of leukemia cells, and c-Myb activation is able to promote the growth of liver cancer.[31,32] However, the mechanisms of tumor resistance induced by MYB are still incompletely elucidated. Particularly, the role of MYB in cisplatin resistance of OCCS has never been demonstrated. In the present study, we showed a relationship between LOC102724169 and MYB on improving the therapeutic efficacy of cisplatin in OCCS. In terms of function, our data revealed that LOC102724169 could improve the cisplatin response rate in ovarian cancer cells (Figures 4C and 4D), even in cisplatin-resistant ovarian cancer cells (Figures S1F and S1G), but this needed more experiments for further exploration. Additionally, MYB was found to be an authenticated target of LOC102724169. Mechanistically, combined with KEGG pathways analysis, our data showed that overexpressed LOC102724169 could decrease MYB by attenuating the PI3K/AKT pathway to enhance cisplatin chemosensitivity and exert an anti-tumor effect in OCCS (Figure 5G). Also, this situation occurred in two types of EOC cell lines: similar results were observed in both nude mice and C57BL/6 mice models. No matter if compared with the NC-ID8/stressed group in C57BL/6 mice or the NC-SKOV3/stressed group in nude mice, the combination of LOC102724169 and cisplatin showed an excellent synergistic anti-tumor effect of more than 80%; this indicates that LOC102724169 could enhance the therapeutic efficacy of cisplatin both *in vivo* and *in vitro*. Overall, our study allowed us to speculate that LOC102724169 might serve as a novel therapeutic target in OCCS complementary to cisplatin. Our findings further documented

Table 3. Univariate and multivariate analysis of clinical-pathological parameters in association with progression-free survival

	Univariate analysis			Multivariate analysis[a]		
Variable	HR	95% CI	p value	HR	95% CI	p value
Age	0.997	0.971–1.024	0.820	0.997	0.964–1.031	0.864
Grade	9.331	2.677–32.516	0.000****	7.353	1.916–28.571	0.004**
FIGO stage	6.579	2.272–19.049	0.001**	3.584	1.185–10.836	0.024*
Lymph node metastasis	3.1	1.543–6.228	0.001**	2.272	1.069–4.829	0.033*
Residual disease	1.963	1.034–3.727	0.039*	2.795	1.135–6.878	0.025*
Cisplatin resistance	4.145	2.132–8.059	0.000****	5.326	1.83–15.15	0.002**
Chronic stress	3.213	1.653–6.242	0.001**	2.659	1.345–5.257	0.005**
Expression of LOC102724169	0.308	0.153–0.621	0.001**	0.309	0.130–0.732	0.008**

HR, hazard ratio; CI, confidence interval. *p < 0.05, **p < 0.01, ***p < 0.001, ****p < 0.0001.
[a]Cox hazard model.

that stress could enhance the pathogenesis of ovarian carcinoma. Furthermore, we characterized the stress-related lncRNA LOC102724169 as a tumor suppressor, which significantly reduced stress-mediated cancer growth of EOC *in vivo* and *in vitro*. Specifically, in proof-of-concept studies, we demonstrated that reintroduction of LOC102724169 expression blocked the PI3K/AKT pathway and therapeutically inhibited MYB. More importantly, there is a strong correlation between LOC102724169 expression and clinical pathology, including residual disease and risks of stress, indicating that LOC102724169 could serve as an effective target for an ovarian cancer theranostic, especially for OCCS. In conclusion, we provide the first evidence that LOC102724169 was able to block the stimulatory effects of chronic stress on cancer growth in the context of primary human ovarian cancer combined with the mouse model, which suggests a promising new curative treatment for ovarian cancer.

MATERIALS AND METHODS

Patients and EOC tissue samples

Patients were recruited from the Department of Gynecologic Oncology, Hunan Cancer Hospital, The Affiliated Cancer Hospital of Xiangya School of Medicine, Central South University (Changsha, P.R. China). Specimens included BOT tissues and EOC tumor tissues. All psycho-social data, as well as tumor tissues, were collected from 86 patients undergoing primary surgical resection of ovarian carcinoma. Benign tumor tissues were obtained from 15 patients with BOTs, and 71 specimens of ovarian cancer were from surgically resected tissues of those ovarian cancer patients. All participants signed informed consent and were reviewed by the Hunan Cancer Hospital Institutional Review Board (no. KYJJ-2018-035). All patients were treated with standard protocols according to the NCCN (National Comprehensive Cancer Network) clinical practice guidelines between 2017 and 2018. The surgical evaluation was used to determine the presence of metastases in accordance with the 2014 FIGO classification.[47] Depressive symptoms were assessed by the depression module of PHQ-9.[26,27]

Cell culture

The human EOC cell lines SKOV3, HO8910, SKOV3/cisplatin, the normal ovarian cell line IOSE80, and the mouse EOC cell line ID8 were purchased from the Type Culture Collection of the Chinese Academy of Sciences (Shanghai, P.R. China). Cell line identification was validated by short tandem repeat profiling and an interspecies contamination test. SKOV3, as well as HO8910, was cultured in Dulbecco's modified Eagle's medium (DMEM, HyClone, USA), while SKOV3/cisplatin, IOSE80, and ID8 cells were cultured in RPMI 1640 (HyClone, USA) added with 1% penicillin-streptomycin solution (HyClone, USA) and 10% fetal bovine serum (FBS, BI, Israel) in a 37°C incubator with 5% CO_2.[48]

Transduction

For lentivirus transduction, SKOV3, HO8910, or ID8 cells were incubated in a 24-well plate with 500 μL of medium containing 20 μL (107 U) LOC102724169 or negative control lentivirus particles and 5 μg/mL Polybrene for 24 h. The liquid was then changed, and puromycin was screened for 21 days to construct stable cell lines (NC-SKOV3/LOC-SKOV3, NC-HO8910/LOC-HO8910, and NC-ID8/LOC-ID8). Plasmid transfections were performed according to the manufacturer's instructions for Lipofectamine 3000 (Invitrogen, USA).

Transcriptome sequencing and analysis

Tumor tissues were removed from an OCCS mouse model and the quality of the RNA was checked by the Agilent 2200 TapeStation system (Agilent Technologies, Santa Clara, CA, USA) and stored at −80°C. RNA sequencing was conducted by Shanghai Novelbio. We applied the DESeq algorithm, using the criteria fold change (FC) >2 or <0.5 and false discovery rate (FDR) <0.05, to screen the differentially expressed mRNAs and lncRNAs. We use a Fisher's exact test for Gene Ontology (GO) analysis based on the genes annotated in the comprehensive GO database. The KEGG pathway enrichment of differential genes was performed by Fisher's exact test.

RNA extraction, reverse transcription, and quantitative real-time PCR

The mRNA level of those differentially expressed genes was accessed by qRT-PCR. Briefly, RNA was isolated with a total RNA TRIzol kit

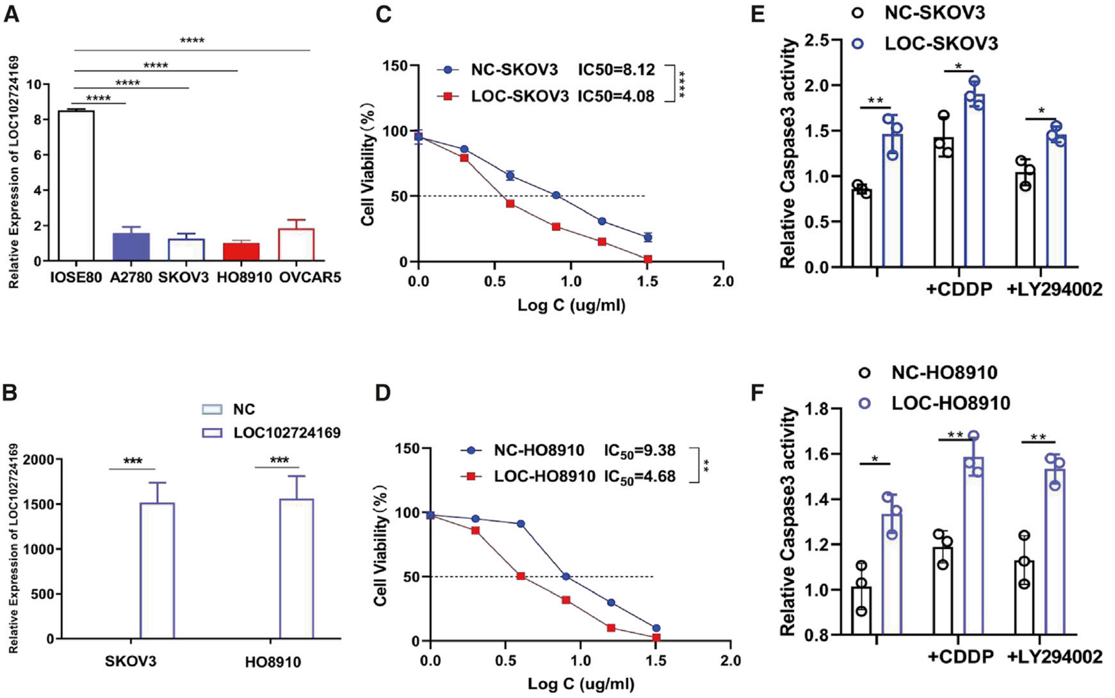

Figure 4. LOC102724169 was necessary for reducing cell viability and inducing apoptosis of EOC cells

(A) Relative expression of LOC102724169 was tested in EOC cell lines (A2780, SKOV3, HO8910, OVCAR5) and the normal ovarian cell line (IOSE80) using qRT-PCR assays. (B) Relative LOC102724169 expression was tested in SKOV3 and HO8910 cells transfected with LOC102724169 transcript or negative control using qRT-PCR assays. (C and D) IC_{50} of SKOV3 and HO8910 cells transfected with LOC102724169 transcript was determined by CCK-8 assays using a wide range of cisplatin concentrations. (E and F) Caspase-3 activity assays indicated the expression of caspase-3 among different groups in both SKOV3 and HO8910 cells. $^{*}p < 0.05$, $^{**}p < 0.01$, $^{***}p < 0.001$, $^{****}p < 0.0001$. Data are represented as mean ± SEM.

(Life Technologies, USA). A RevertAid first-strand cDNA synthesis kit (Thermo Fisher Scientific, USA) was used to synthesize cDNA. qRT-PCR was then carried out using ChamQ SYBR color qPCR master mix (Vazyme, P.R. China) normalized to GAPDH levels, and the data were analyzed using the $\Delta\Delta$Ct method.[49] The primer sequences used in qRT-PCR are presented in Table S1.

Cell viability assay

Cells were seeded in a 96-well plate at a density of 5×10^3 cells/well (100 μL). After treating these cancer cells with different concentrations of cisplatin for 24 h, cell viability was evaluated using a CCK-8 (Vazyme, P.R. China). The absorbance was measured by a plate reader (Bio-Rad, Hercules, CA, USA) at 450 nm.

Migration and invasion assay

Transwell systems (Corning Life Sciences, USA) were used to evaluate the metastatic ability of cancer cells. Briefly, for the invasion assay, Matrigel (BD Biosciences, USA) was added to the upper surface of a polycarbonate membrane (pore size 8 μm) to form a thin gel layer to serve as the extracellular matrix (ECM). The upper compartment of the filter contained the treated cells at a density of 5×10^5 cells/well in 200 μL of DMEM. The bottom filter was filled with 600 μL of conditioned medium. After incubation at 37°C with 5% CO_2 for 48 h, the polycarbonate membrane was then fixed with 4% paraformaldehyde for 10 min and stained with 0.2% crystal violet solution. Then, the cells on the upper surface of the filter were erased by wiping with a cotton swab. Cells that penetrated to the lower surface were counted under an Olympus microscope in three randomized fields at a magnification of ×100/×200. A cell migration assay was carried out in a transwell filter on membrane filters without Matrigel.

Dual-luciferase reporter assay system

Luciferase reporter assays were performed to detect the direct binding of CEBPB to the promoter region of LOC102724169. The +2,000-bp region upstream of LOC102724169 is considered as its promoter region. The entire promoter region of LOC102724169 was cloned into the pGL3-basic vector (Tsingke, Shanghai, P.R. China). To test the binding specificity, the sequences of CEBPB that bound with the LOC102724169 promoter region were created (BP1, 5′-AGTTTCAACAC-3′; BP2, 5′-TATTTTATAAT-3′; BP3, 5′-TATTTCACCAC-3′), and they were also inserted into a pENTER-CEBPB plasmid and EV-pENTER plasmids. Briefly, SKOV3 cells, as well as HO8910 cells, were seeded in a 24-well plate containing antibiotic-free media. Cells were co-transfected with the LOC102724169 promoter construct with either pENTER-CEBPB or EV-pENTER (NC) plasmid using Lipofectamine 3000. PRL-TK plasmid (Tsingke, Shanghai, P.R. China) was also transfected into each group and served as an internal control

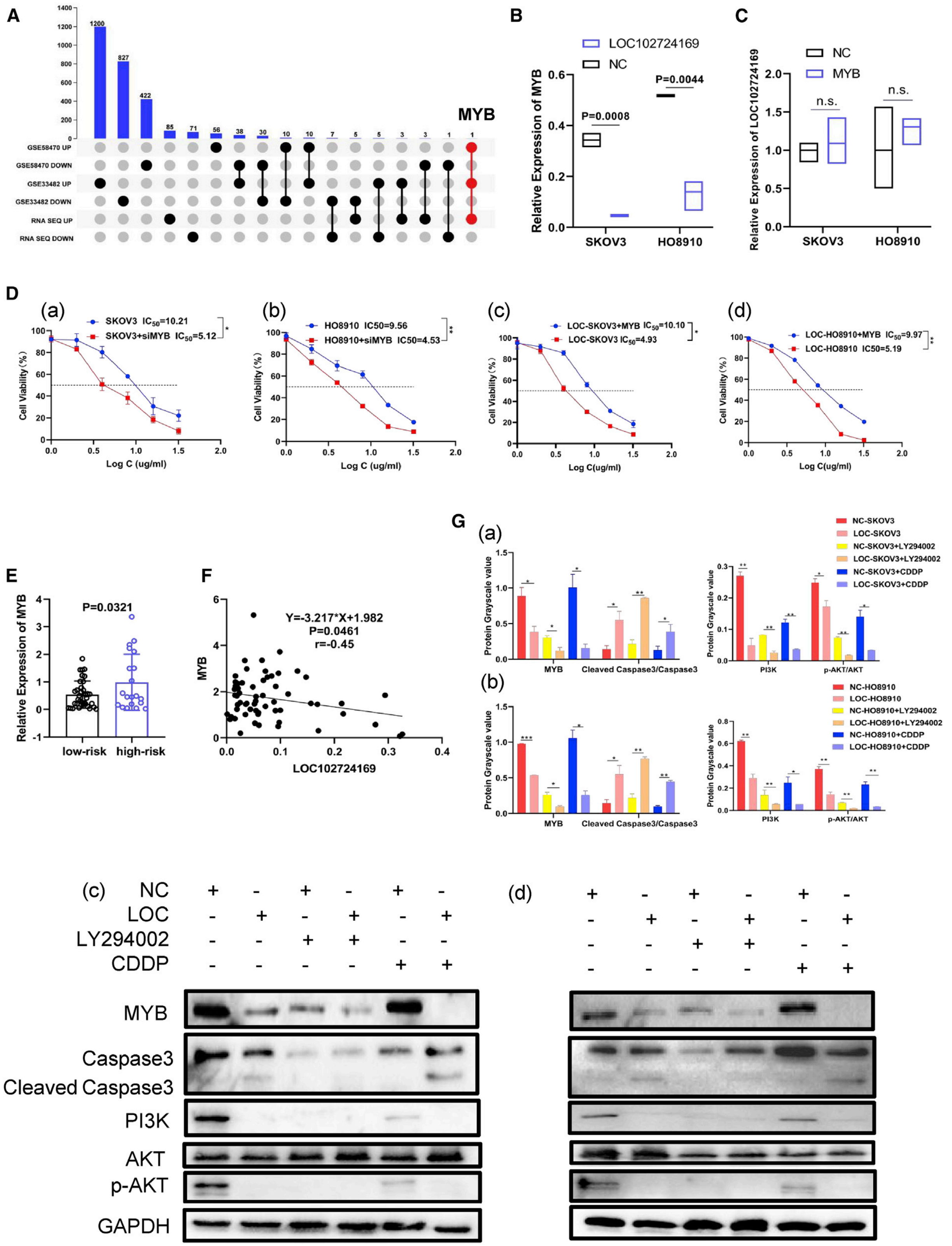

(legend on next page)

reporter. After transfection for 48 h, cells were collected and analyzed using the Dual-Glo luciferase assay system (Promega, USA). Luciferase activity was measured with an M200 microplate fluorescence reader (Tecan, Beijing, P.R. China) and the results were normalized to Renilla luciferase activity. Transfection experiments were duplicated and repeated in at least three independent experiments.

Western blot

After lysing in radioimmunoprecipitation assay (RIPA) buffer (Vazyme, P.R. China), which contained protease inhibitors (Roche, Germany), cells were washed with D-Hanks solution. Proteins were quantified with the bicinchoninic acid (BCA) protein assay kit (Vazyme, P.R. China). Samples containing 30 μg of total proteins were electrophoresed on SDS-polyacrylamide gels and then transferred onto a polyvinylidene fluoride (PVDF) membrane (Millipore, USA) by electroblotting using a Bio-Rad Bis-Tris gel system (Bio-Rad, USA). The membranes that were covered with the primary antibody were blocked by 5% nonfat milk and incubated overnight at 4°C, then washed with Tris-buffered saline with Tween 20 (TBST), and subsequently incubated with a secondary antibody for 1 h at 37°C. Finally, an enhanced chemiluminescence (ECL) solution (Millipore, USA) was added to cover the blotting surface. The signals were captured, and the intensity of the bands was quantified by using the Bio-Rad ChemiDoc XRS+ system (Bio-Rad, USA). Proteins isolated from tissues and cells were incubated with a primary antibody that included MYB (Abcam, USA), GAPDH (SAB, USA), caspase-3, AKT, phosphorylated (p-)AKT (Ser473), PI3K (Cell Signaling Technology, MA, USA).

ChIP assay

Briefly, SKOV3 and HO8910 cells were crosslinked in 1% formaldehyde for 10 min at 37°C. DNA from fixed chromatin cells was then subjected to immunoprecipitation using a ChIP assay kit (Millipore, Billerica, MA, USA) and the antibodies against CEBPB (Thermo Fisher Scientific, USA) or anti-rabbit immunoglobulin G (IgG) (Santa Cruz Biotechnology, Santa Cruz, CA, USA) according to the manufacturers' protocol. The precipitated DNA fragments were purified and measured by qPCR under the conditions described above. Primers specific to each segment of interest are listed as follows: LOC102724169-pro (BP1), forward, 5′-TATTTTTAAGTGGATGT GGTAG-3′ and reverse, 5′-CGTTCTAAGCTCTTTCAGTCC-3′; LOC102724169-pro (BP2), forward, 5′-GAGGTGGCGTGTGGAA GGC-3′ and reverse, 5′-GGTCTGGGTAGCAGATGATGC-3′; LOC 102724169-pro (BP3), forward, 5′-TTCTTGAATGACACTGTGA TA-3′ and reverse, 5′-AAGGACATTAGTTTTTGGTAC-3′.

Caspase-3 activity assay

A caspase-3 activity kit (Solarbio, Beijing, P.R.China) was used for the determination of caspase-3 activity in cell lysates following the manufacturer's protocol.

IHC

The specimens were paraffin-embedded, after which the tissue sections (3 μm) were dewaxed, rehydrated, and blocked with 3% BSA and subjected to antigen retrieval. After being washed, the sections were incubated against CEBPB antibodies (Cell Signaling Technology, St. Louis, MO, USA) at 4°C overnight. Mouse IgG was then used as the negative control. After being washed again, the bound antibodies were detected using horseradish peroxidase (HRP)-conjugated goat anti-rabbit IgG and goat anti-mouse IgG and visualized by diaminobenzidine (DAB). The results were graded according to both the intensity and the percentage of positive cells under a microscope by two pathologists in a blinded manner.

ISH

ISH was performed to detect the location of LOC102724169. The probe LOC102724169 was designed by Boster (Wuhan, P.R. China). RNA ISH was performed with an ISH detection kit (Boster) according to the manufacturer's instructions.

Animal model

The female nude mice and C57BL/6 mice, 5–7 weeks old, were purchased from Hunan SJA Laboratory Animal Co. (SJA, P.R. China). Mice were subjected to a restraint-stress procedure based on the previous study.[3] The physical restraint systems were transparent perforated 50-mL centrifuge tubes. Sterilization of the experimental equipment was conducted daily.

For a single OCCS mouse model, nude mice were housed and randomly divided into two groups: control and daily stress. We injected 5×10^6 SKOV3 cells/200 μL/mouse into the peritoneal cavity of nude mice after a week of chronic stress. All mice were euthanized after restraint stress for 35 days. The tumor was removed for high-throughput sequencing and the blood was also collected and centrifuged for further study.

For the LOC102724169-treated OCCS mouse model, after tumorigenesis of the single CSOC model constructed in the SKOV3 cell line, we injected overexpression of LOC102724169 or empty vector viruses into the peritoneal cavity (5×10^8 plaque-forming unit [PFU] viruses per nude mice). Alternatively, 1×10^7 NC-ID8 or

Figure 5. LOC102724169 regulated the expression of MYB and affected the PI3K/AKT pathway in OCCS

(A) Schematic overview of the workflow used to investigate the target gene of LOC102724169 in OCCS. (B) Relative expression of MYB in both SKOV3 and HO8910 cells transfected with LOC102724169 or control plasmid. (C) Relative expression of LOC102724169 in both SKOV3 and HO8910 cells transfected with MYB or control plasmid. (Da and Db) Knockdown of MYB decreased the IC_{50} values of cisplatin in SKOV3 and HO8910 cells. (Dc and Dd) Overexpression of MYB in LOC-SKOV3/HO8910 cells increased the IC_{50} values of cisplatin. (E) Validation of MYB expression between the high-risk stress group and low-risk stress group. (F) Correlation analysis of MYB and LOC102724169 expression levels by a Pearson correlation coefficient ($r = -0.45$, $p = 0.0461$). (Ga and Gb) Histograms represent the quantification of western blot bands in both SKOV3 and HO8910 cells. (Gc and Gd) Western blot analysis of MYB, caspase-3, and PI3K/AKT signaling-related molecule expression in different groups in both SKOV3 and HO8910 cells. $^*p < 0.05$, $^{**}p < 0.01$, $^{***}p < 0.001$. Data are represented as mean ± SEM.

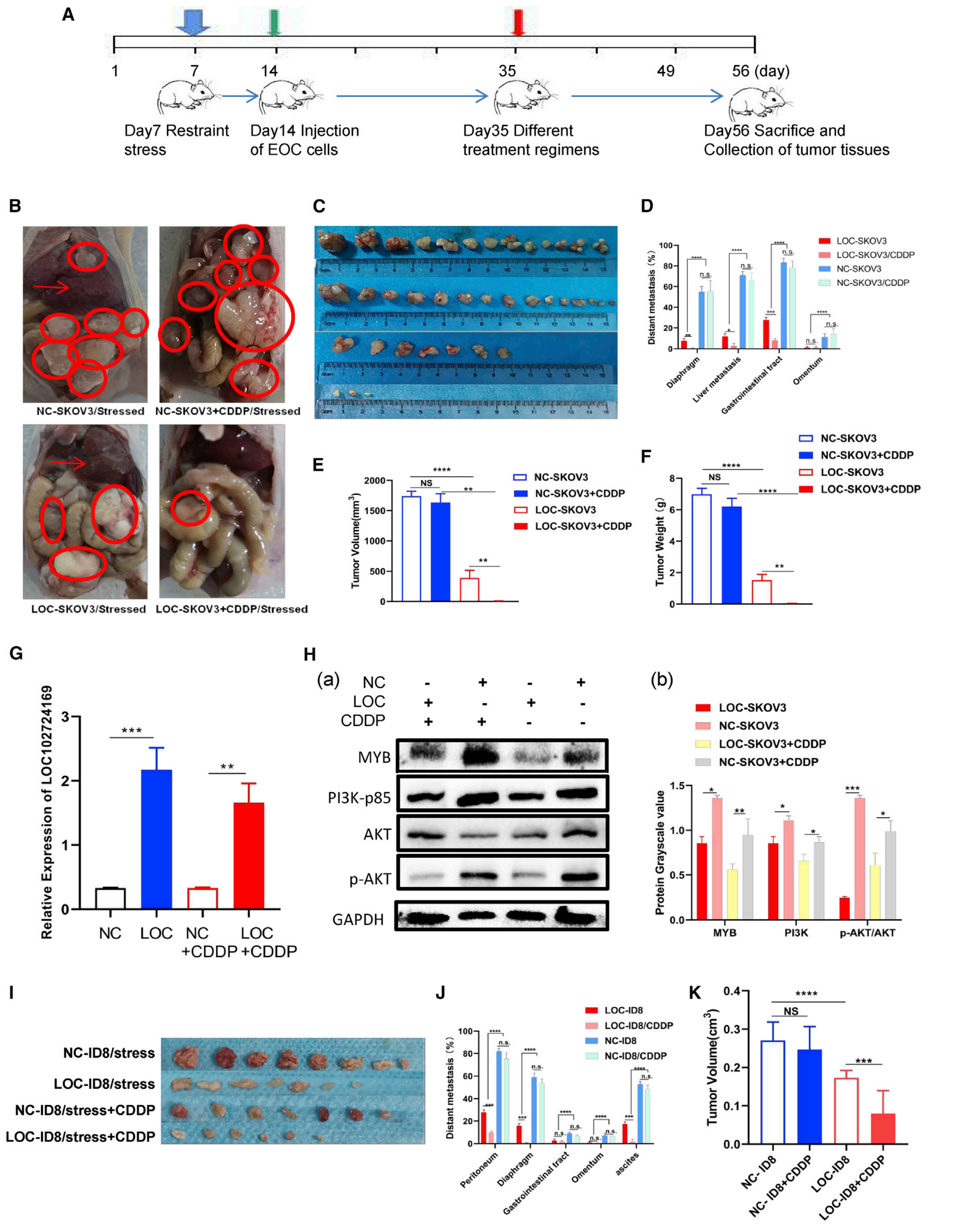

(legend on next page)

LOC-ID8 cells/200 μL/mouse were injected into the peritoneal cavity of C57BL/6 mice. In the cisplatin treatment groups, 4 mg/kg cisplatin was injected through the tail vein every week. The experimental protocol was approved by the Experimental Animal Ethics Review Committee of Hunan Cancer Hospital.

Bioinformatics analysis

We have uploaded our RNA sequencing (RNA-seq) results into GEO: GSE143237. We also downloaded gene expression profiles of the cisplatin resistance dataset from GEO: GSE33482 and GSE58470.[29] All and subsequent analyses were conducted on the above datasets after regression calculation of the datasets by the affyPLM package in R language. The relative logarithmic expression, normalized unscaled standard errors, and RNA degradation were detected. The robust multi-array average (RMA) algorithm was used for background correcting, normalizing, and calculating the expression of the datasets. The probe ID was converted to gene symbol, and k-nearest neighbor complemented the missing value. The limma package calculated the differentially expressed gene (logFC >1 or logFC less than −1, adjusted p value [adj.p.val.] <0.05).[50] Based on gene expression, correlations between genes were calculated using the cor.test() function involved in the psychology package.

Statistical analysis

All quantitative data are expressed as mean ± SEM of three independent experiments unless otherwise indicated. Data from the two groups were compared using the Student's t test. The correlation was analyzed using Spearman rank regression and Pearson correlation. Cumulative survival was calculated by Kaplan-Meier analysis, and the significance of the difference in survival was analyzed by the Gehan-Breslow-Wilcoxon test. Results were considered statistically significant if $p < 0.05$. All data were statistically analyzed with GraphPad Prism8 (GraphPad, La Jolla, CA, USA).

Ethics approval and consent to participate

This study was approved by the Institutional Ethics Committee of Hunan Cancer Hospital/The Affiliated Tumor Hospital of Xiangya School of Medicine, Central South University (no. KYJJ-2018-035). Informed consent was obtained from all patients prior to analysis. All animal experimental methods involved in this study are in line with the Declaration of Helsinki and approved by the Institutional Animal Care and Use Committee of the Affiliated Tumor Hospital of Xiangya School of Medicine, Central South University.

Availability of data and material

Data of high-throughput sequencing are deposited publically in GEO: GSE143237. The additional datasets used were previously published under GEO: GSE33482 and GSE58470.[29]

SUPPLEMENTAL INFORMATION

Supplemental information can be found online at https://doi.org/10.1016/j.omtn.2021.03.001.

ACKNOWLEDGMENTS

We are grateful to all of the technical support from Dr. Jian Ma in the Cancer Invasion and Metastasis Laboratory of the Cancer Research Institute of Xiangya School of Medicine, Central South University, and the key experiments of non-controllable inflammation and tumors in Hunan Province, as well as from the staff in the Central Laboratory of Hunan Cancer Hospital/The Affiliated Cancer Hospital of Xiangya School of Medicine, Central South University. We thank all of our patients for participating in this study. This work was supported by grants from the Guidance Project of Clinical Medical Technology Innovation of Hunan Province (2020SK51101); the General Project of Natural Science Foundation of Hunan Province (no. 2020JJ4051); the Promotion Project of Health Suitability Program in Health Department of Hunan Province (WZ2020-15); the National Cancer Center Climbing Fund (no. NCC2018B61); the National Natural Science Foundation of China (no. 81101996); and by the Hunan Cancer Hospital Climb Plan.

AUTHOR CONTRIBUTIONS

J.T., X.Z., and L.C. conceived and designed the experiments; X.Z., M.L., G.D., and L.S. performed the experiments; X.Z. and M.L. analyzed the data; X.Z., M.L., and G.D. collected the clinical samples and analyzed the clinical data; X.Z. and G.D. performed the bioinformatics and statistical analyses; J.T., X.Z., M.L., and L.C. obtained funds; Y.Z. and L.C. provided technique support; and X.Z. and J.T. drafted and revised the manuscript. All authors gave final approval of the manuscript.

Figure 6. LOC102724169 overexpression increased response rate to cisplatin (CDDP) in the OCCS mice model.

(A) Schematic overview of different treatment regimens on stressed mice. Blue arrow indicates all mice kept for 2 h of immobilization daily during the whole experimental cycle. Green arrow indicates that SKOV3/ID8 cells were injected intraperitoneally in nude mice with periodic immobilization in nude mice/C57BL/6. Red arrow indicates after injection of EOC cells intraperitoneally, all stressed mice were randomly divided into four groups according to different treatments: (1) empty vector viruses (NC-virs/NC-SKOV3), (2) empty vector viruses plus CDDP (NC-virs/NC-SKOV3+CDDP), (3) overexpression of LOC102724169 viruses (LOC-virs/LOC-SKOV3), (4) overexpression of LOC102724169 viruses plus CDDP (LOC-virs/LOC-SKOV3+CDDP). (B) Macroscopic tumor tissue and metastases lesions of four groups are presented, respectively. Red circles mark tumor tissues; red arrows indicate liver metastasis. (C) The tumor tissues of those groups were presented. From top to bottom are NC-SKOV3, NC-SKOV3+CDDP, LOC-SKOV3, and LOC-SKOV3+CDDP. (D) Histogram showed different metastases lesions of four groups. (E) Quantification of tumor volume in four groups. (F) Quantification of tumor weight in four groups. (G) Expression of LOC102724169 was validated in mice tumor tissue in different groups. (Ha) Western blot analysis of MYB and other related molecule expressions in different groups were validated. (Hb) Histogram represents the quantification of bands. (I) NC-ID8/ LOC-ID8 cells were intraperitoneally injected into C57BL/6 mice. The tumor tissues of NC/LOC-ID8 and those treated with cisplatin groups are presented. (J) Histogram shows different metastasis lesions of four groups in C57BL/6 mice. (K) Quantification of tumor volume in four groups. $^{*}p < 0.05$, $^{**}p < 0.01$, $^{***}p < 0.001$, $^{****}p < 0.0001$. Statistical differences calculated using a two-tailed Student's t test. Data are represented as mean ± SEM.

DECLARATION OF INTERESTS

The authors declare no competing interests.

REFERENCES

1. Jayson, G.C., Kohn, E.C., Kitchener, H.C., and Ledermann, J.A. (2014). Ovarian cancer. Lancet *384*, 1376–1388.
2. Bast, R.C., Jr., Hennessy, B., and Mills, G.B. (2009). The biology of ovarian cancer: New opportunities for translation. Nat. Rev. Cancer *9*, 415–428.
3. Thaker, P.H., Han, L.Y., Kamat, A.A., Arevalo, J.M., Takahashi, R., Lu, C., Jennings, N.B., Armaiz-Pena, G., Bankson, J.A., Ravoori, M., et al. (2006). Chronic stress promotes tumor growth and angiogenesis in a mouse model of ovarian carcinoma. Nat. Med. *12*, 939–944.
4. Lutgendorf, S.K., DeGeest, K., Sung, C.Y., Arevalo, J.M., Penedo, F., Lucci, J., 3rd, Goodheart, M., Lubaroff, D., Farley, D.M., Sood, A.K., and Cole, S.W. (2009). Depression, social support, and beta-adrenergic transcription control in human ovarian cancer. Brain Behav. Immun. *23*, 176–183.
5. Idahl, A., Hermansson, A., and Lalos, A. (2018). Social support and ovarian cancer incidence—A Swedish prospective population-based study. Gynecol. Oncol. *149*, 324–328.
6. Scarpa, J.R., Jr., Fatma, M., Loh, Y.E., Traore, S.R., Stefan, T., Chen, T.H., Nestler, E.J., and Labonté, B. (2020). Shared transcriptional signatures in major depressive disorder and mouse chronic stress models. Biol. Psychiatry *88*, 159–168.
7. Antoni, M.H., Lutgendorf, S.K., Cole, S.W., Dhabhar, F.S., Sephton, S.E., McDonald, P.G., Stefanek, M., and Sood, A.K. (2006). The influence of bio-behavioural factors on tumour biology: Pathways and mechanisms. Nat. Rev. Cancer *6*, 240–248.
8. Powell, N.D., Tarr, A.J., and Sheridan, J.F. (2013). Psychosocial stress and inflammation in cancer. Brain Behav. Immun. *30* (*Suppl*), S41–S47.
9. Nilsson, M.B., Armaiz-Pena, G., Takahashi, R., Lin, Y.G., Trevino, J., Li, Y., Jennings, N., Arevalo, J., Lutgendorf, S.K., Gallick, G.E., et al. (2007). Stress hormones regulate interleukin-6 expression by human ovarian carcinoma cells through a Src-dependent mechanism. J. Biol. Chem. *282*, 29919–29926.
10. Watkins, J.L., Thaker, P.H., Nick, A.M., Ramondetta, L.M., Kumar, S., Urbauer, D.L., Matsuo, K., Squires, K.C., Coleman, R.L., Lutgendorf, S.K., et al. (2015). Clinical impact of selective and nonselective beta-blockers on survival in patients with ovarian cancer. Cancer *121*, 3444–3451.
11. Baek, M.H., Kim, D.Y., Kim, S.O., Kim, Y.J., and Park, Y.H. (2018). Impact of beta blockers on survival outcomes in ovarian cancer: a nationwide population-based cohort study. J. Gynecol. Oncol. *29*, e82.
12. Barron, T.I., Connolly, R.M., Sharp, L., Bennett, K., and Visvanathan, K. (2011). Beta blockers and breast cancer mortality: A population-based study. J. Clin. Oncol. *29*, 2635–2644.
13. Lemeshow, S., Sørensen, H.T., Phillips, G., Yang, E.V., Antonsen, S., Riis, A.H., Lesinski, G.B., Jackson, R., and Glaser, R. (2011). β-Blockers and survival among Danish patients with malignant melanoma: A population-based cohort study. Cancer Epidemiol. Biomarkers Prev. *20*, 2273–2279.
14. Heitz, F., Hengsbach, A., Harter, P., Traut, A., Ataseven, B., Schneider, S., Prader, S., Kurzeder, C., Sporkmann, M., and du Bois, A. (2017). Intake of selective beta blockers has no impact on survival in patients with epithelial ovarian cancer. Gynecol. Oncol. *144*, 181–186.
15. Thaker, P.H., Urbauer, D.L., and Sood, A.K. (2016). Reply to beta blockers in epithelial ovarian cancer and beta-blockers and improved survival from ovarian cancer: New miracle treatment or another case of immortal person-time bias? Cancer *122*, 325–326.
16. Schmidt, S.A., and Schmidt, M. (2016). Beta-blockers and improved survival from ovarian cancer: New miracle treatment or another case of immortal person-time bias? Cancer *122*, 324–325.
17. Iyer, M.K., Niknafs, Y.S., Malik, R., Singhal, U., Sahu, A., Hosono, Y., Barrette, T.R., Prensner, J.R., Evans, J.R., Zhao, S., et al. (2015). The landscape of long noncoding RNAs in the human transcriptome. Nat. Genet. *47*, 199–208.
18. Huarte, M. (2015). The emerging role of lncRNAs in cancer. Nat. Med. *21*, 1253–1261.
19. Tripathi, M.K., Doxtater, K., Keramatnia, F., Zacheaus, C., Yallapu, M.M., Jaggi, M., and Chauhan, S.C. (2018). Role of lncRNAs in ovarian cancer: defining new biomarkers for therapeutic purposes. Drug Discov. Today *23*, 1635–1643.
20. Zheng, Z.G., Xu, H., Suo, S.S., Xu, X.L., Ni, M.W., Gu, L.H., Chen, W., Wang, L.Y., Zhao, Y., Tian, B., and Hua, Y.J. (2016). The essential role of H19 contributing to cisplatin resistance by regulating glutathione metabolism in high-grade serous ovarian cancer. Sci. Rep. *6*, 26093–26105.
21. Jin, Y., Feng, S.J., Qiu, S., Shao, N., and Zheng, J.H. (2017). ncRNA MALAT1 promotes proliferation and metastasis in epithelial ovarian cancer via the PI3K-AKT pathway. Eur. Rev. Med. Pharmacol. Sci. *21*, 3176–3184.
22. Long, X., Song, K., Hu, H., Tian, Q., Wang, W., Dong, Q., Yin, X., and Di, W. (2019). Long non-coding RNA GAS5 inhibits DDP-resistance and tumor progression of epithelial ovarian cancer via GAS5-E2F4-PARP1-MAPK axis. J. Exp. Clin. Cancer Res. *38*, 345–360.
23. Richards, E.J., Permuth-Wey, J., Li, Y., Chen, Y.A., Coppola, D., Reid, B.M., Lin, H.Y., Teer, J.K., Berchuck, A., Birrer, M.J., et al. (2015). A functional variant in *HOXA11-AS*, a novel long non-coding RNA, inhibits the oncogenic phenotype of epithelial ovarian cancer. Oncotarget *6*, 34745–34757.
24. Qu, L., Ding, J., Chen, C., Wu, Z.J., Liu, B., Gao, Y., Chen, W., Liu, F., Sun, W., Li, X.F., et al. (2016). Exosome-transmitted lncARSR promotes sunitinib resistance in renal cancer by acting as a competing endogenous RNA. Cancer Cell *29*, 653–668.
25. Gong, C., Li, Z., Ramanujan, K., Clay, I., Zhang, Y., Lemire-Brachat, S., and Glass, D.J. (2015). A long non-coding RNA, *LncMyoD*, regulates skeletal muscle differentiation by blocking IMP2-mediated mRNA translation. Dev. Cell *34*, 181–191.
26. Kroenke, K., Spitzer, R.L., and Williams, J.B. (2001). The PHQ-9: Validity of a brief depression severity measure. J. Gen. Intern. Med. *16*, 606–613.
27. Spitzer, R.L., Kroenke, K., and Williams, J.B.; Primary Care Evaluation of Mental Disorders (1999). Validation and utility of a self-report version of PRIME-MD: The PHQ primary care study. Primary Care Evaluation of Mental Disorders. Patient health questionnaire. JAMA *282*, 1737–1744.
28. Lutgendorf, S.K., Shinn, E., Carter, J., Leighton, S., Baggerly, K., Guindani, M., Fellman, B., Matzo, M., Slavich, G.M., Goodman, M.T., et al. (2017). Quality of life among long-term survivors of advanced stage ovarian cancer: A cross-sectional approach. Gynecol. Oncol. *146*, 101–108.
29. Arrighetti, N., Cossa, G., De Cecco, L., Stucchi, S., Carenini, N., Corna, E., Gandellini, P., Zaffaroni, N., Perego, P., and Gatti, L. (2016). PKC-alpha modulation by *miR-483-3p* in platinum-resistant ovarian carcinoma cells. Toxicol. Appl. Pharmacol. *310*, 9–19.
30. Stenman, G., Andersson, M.K., and Andrén, Y. (2010). New tricks from an old oncogene: Gene fusion and copy number alterations of MYB in human cancer. Cell Cycle *9*, 2986–2995.
31. Wang, Y., Fang, R., Cui, M., Zhang, W., Bai, X., Wang, H., Liu, B., Zhang, X., and Ye, L. (2017). The oncoprotein HBXIP up-regulates YAP through activation of transcription factor c-Myb to promote growth of liver cancer. Cancer Lett. *385*, 234–242.
32. Nakano, K., Uchimaru, K., Utsunomiya, A., Yamaguchi, K., and Watanabe, T. (2016). Dysregulation of c-Myb pathway by aberrant expression of proto-oncogene MYB provides the basis for malignancy in adult T-cell leukemia/lymphoma cells. Clin. Cancer Res. *22*, 5915–5928.
33. Chen, H., Liu, D., Guo, L., Cheng, X., Guo, N., and Shi, M. (2018). Chronic psychological stress promotes lung metastatic colonization of circulating breast cancer cells by decorating a pre-metastatic niche through activating β-adrenergic signaling. J. Pathol. *244*, 49–60.
34. Jiang, W., Li, Y., Li, Z.Z., Sun, J., Li, J.W., Wei, W., Li, L., Zhang, C., Huang, C., Yang, S.Y., et al. (2019). Chronic restraint stress promotes hepatocellular carcinoma growth by mobilizing splenic myeloid cells through activating β-adrenergic signaling. Brain Behav. Immun. *80*, 825–838.
35. Xie, H., Li, C., He, Y., Griffin, R., Ye, Q., and Li, L. (2015). Chronic stress promotes oral cancer growth and angiogenesis with increased circulating catecholamine and glucocorticoid levels in a mouse model. Oral Oncol. *51*, 991–997.
36. Lutgendorf, S.K., Sood, A.K., Anderson, B., McGinn, S., Maiseri, H., Dao, M., Sorosky, J.I., De Geest, K., Ritchie, J., and Lubaroff, D.M. (2005). Social support, psychological distress, and natural killer cell activity in ovarian cancer. J. Clin. Oncol. *23*, 7105–7113.

37. Glaser, R., and Kiecolt-Glaser, J.K. (2005). Stress-induced immune dysfunction: Implications for health. Nat. Rev. Immunol. *5*, 243–251.

38. Shahabi, S., Kumaran, V., Castillo, J., Cong, Z., Nandagopal, G., Mullen, D.J., Alvarado, A., Correa, M.R., Saizan, A., Goel, R., et al. (2019). *LINC00261* is an epigenetically regulated tumor suppressor essential for activation of the DNA damage response. Cancer Res. *79*, 3050–3062.

39. Lu, Y., Hu, Z., Mangala, L.S., Stine, Z.E., Hu, X., Jiang, D., Xiang, Y., Zhang, Y., Pradeep, S., Rodriguez-Aguayo, C., et al. (2018). MYC targeted long noncoding RNA DANCR promotes cancer in part by reducing p21 levels. Cancer Res. *78*, 64–74.

40. Johansson, J., Berg, T., Kurzejamska, E., Pang, M.F., Tabor, V., Jansson, M., Roswall, P., Pietras, K., Sund, M., Religa, P., and Fuxe, J. (2013). miR-155-mediated loss of C/EBPβ shifts the TGF-β response from growth inhibition to epithelial-mesenchymal transition, invasion and metastasis in breast cancer. Oncogene *32*, 5614–5624.

41. Wu, Y.H., Chang, T.H., Huang, Y.F., Chen, C.C., and Chou, C.Y. (2015). COL11A1 confers chemoresistance on ovarian cancer cells through the activation of Akt/c/EBPβ pathway and PDK1 stabilization. Oncotarget *6*, 23748–23763.

42. Lu, J., Chen, W., Liu, H., Yang, H., and Liu, T. (2019). Transcription factor CEBPB inhibits the proliferation of osteosarcoma by regulating downstream target gene CLEC5A. J. Clin. Lab. Anal. *33*, e22985.

43. Martin, L., and Schilder, R. (2007). Novel approaches in advancing the treatment of epithelial ovarian cancer: the role of angiogenesis inhibition. J. Clin. Oncol. *25*, 2894–2901.

44. Pujade-Lauraine, E., Hilpert, F., Weber, B., Reuss, A., Poveda, A., Kristensen, G., Sorio, R., Vergote, I., Witteveen, P., Bamias, A., et al. (2014). Bevacizumab combined with chemotherapy for platinum-resistant recurrent ovarian cancer: The AURELIA open-label randomized phase III trial. J. Clin. Oncol. *32*, 1302–1308.

45. Wei, L., Wang, X., Lv, L., Liu, J., Xing, H., Song, Y., Xie, M., Lei, T., Zhang, N., and Yang, M. (2019). The emerging role of microRNAs and long noncoding RNAs in drug resistance of hepatocellular carcinoma. Mol. Cancer *18*, 147.

46. Smallegan, M.J., and Rinn, J.L. (2019). Linking long noncoding RNA to drug resistance. Proc. Natl. Acad. Sci. USA *116*, 21963–21965.

47. Zeppernick, F., and Meinhold-Heerlein, I. (2014). The new FIGO staging system for ovarian, fallopian tube, and primary peritoneal cancer. Arch. Gynecol. Obstet. *290*, 839–842.

48. Chen, L., Yao, Y., Sun, L., Zhou, J., Liu, J., Wang, J., Li, J., and Tang, J. (2015). Clinical implication of the serum galectin-1 expression in epithelial ovarian cancer patients. J. Ovarian Res. *8*, 78–89.

49. Chen, L., Yao, Y., Sun, L., and Tang, J. (2017). Galectin-1 promotes tumor progression via NF-κB signaling pathway in epithelial ovarian cancer. J. Cancer *8*, 3733–3741.

50. Zhao, L., Wang, W., Xu, L., Yi, T., Zhao, X., Wei, Y., Vermeulen, L., Goel, A., Zhou, S., and Wang, X. (2019). Integrative network biology analysis identifies miR-508-3p as the determinant for the mesenchymal identity and a strong prognostic biomarker of ovarian cancer. Oncogene *38*, 2305–2319.

OMTN, Volume *24*

Supplemental information

lncRNA LOC102724169 plus cisplatin exhibit the synergistic anti-tumor effect in ovarian cancer with chronic stress

Xiaofang Zhou, Mu Liu, Guanming Deng, Le Chen, Lijuan Sun, Yun Zhang, Chenhui Luo, and Jie Tang

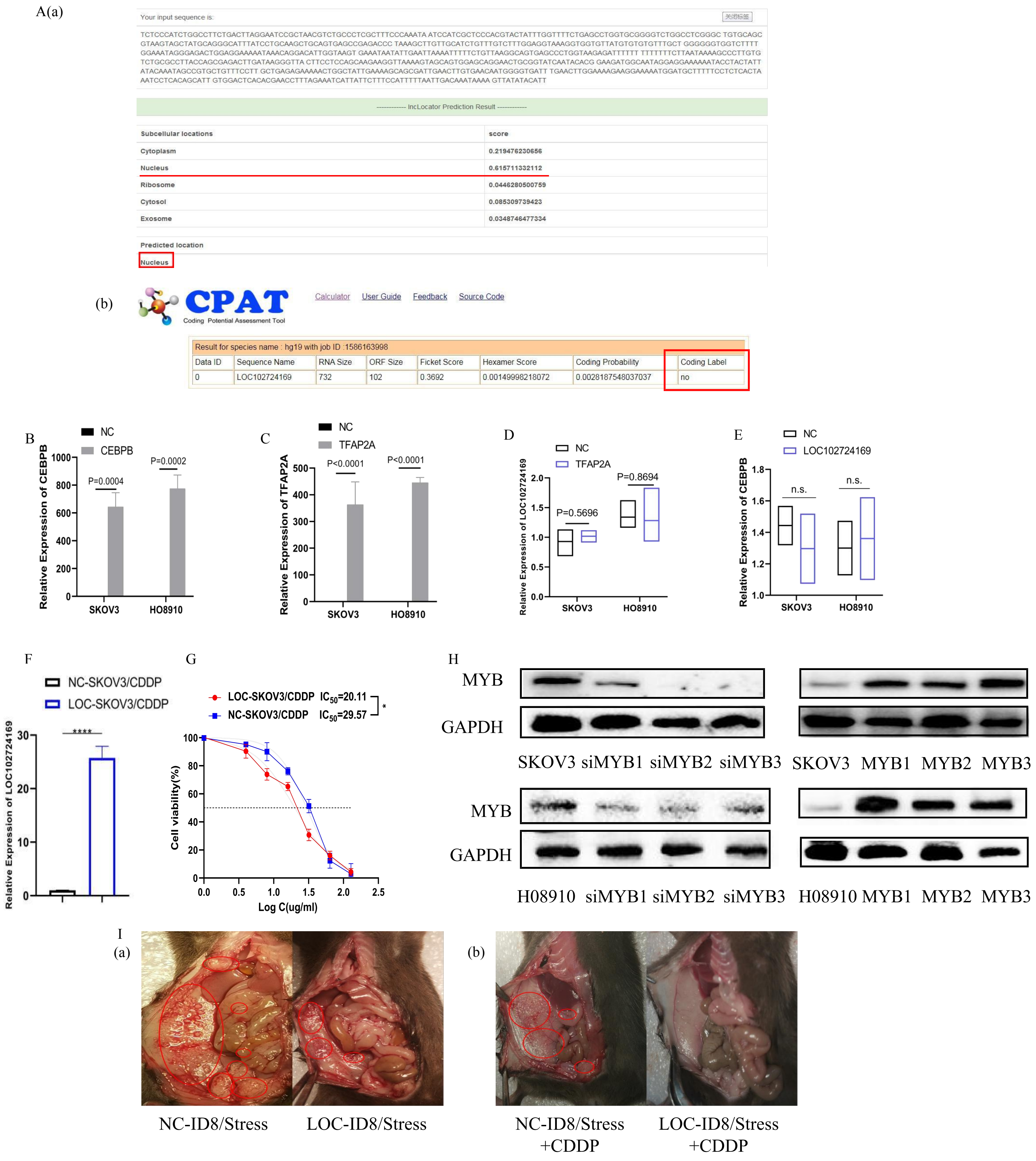

Supplementary Figure 1: A(a): lncLocator (http://www.csbio.sjtu.edu.cn/cgi-bin/lncLocator.py) suggested that LOC102724169 was mostly located in the cell nucleus. (b): A very low coding potential of LOC102724169 was predicted by CPC (coding potential calculator, http://cpc.cbi.pku.edu.cn/). B: Relative CEBPB expression in SKOV3 and HO8910 cells transfected with CEBPB expression vector or negative control, measured using RT-qPCR and normalized to GAPDH. C: Relative TFAP2A expression in SKOV3 and HO8910 cells transfected with CEBPB expression vector or negative control, measured using RT-qPCR and normalized to GAPDH. D: Relative expression of LOC102724169 in both SKOV3 and HO8910 cells transfected with CEBPB or control plasmid. E: Relative expression of CEBPB in both SKOV3 and HO8910 cells transfected with LOC102724169 or control plasmid. F: Relative expression of LOC102724169 in SKOV3/CDDP cells transfected with LOC102724169 or control plasmid. G: IC50 of SKOV3/CDDP cells transfected with LOC102724169 transcript was determined by CCK8 assays using a wide range of cisplatin concentrations. H: Relative MYB expression after SKOV3 and HO8910 cells were transfected with siRNAs targeting MYB (left panel) or MYB plasmids (right panel). I(a): NC/LOC-ID8 cells were injected intraperitoneally in stressed C57BL/6 mice respectively. Red circles marked macroscopic tumor tissue and metastases lesions. (b): LOC-ID8/stressed group and NC-ID8/stressed group treated with CDDP. Red circles marked macroscopic tumor tissue and metastases lesions.*$P<0.05$, **$P<0.01$. Statistical differences calculated using two-tailed student' s t-test. Data are represented as mean±SEM. CDDP: (Cisplatin).

Supplemental Table 1: PCR primer sequences

Gene official name	Forward primer	Reverse primer
GAPDH	AACGGATTTGGTCGTATTGG	TTGATTTTGGAGGGATCTCG
LOC102724169	TAAGGCAGTGAGCCCTGGTA	TCAAGTCTCGCTGGTAAGGC
HOTAIRM1	GGACGAATCGCATCCAGGAG	GCCCCTTCCTCCGCTAAATC
AKAP12	GAGATGGCTACTAAGTCAGCGG	CAGTGGGTTGTGTTAGCTCTTC
ARMC9	GAAGCTGAACGTAGGTCAGTG	CAGACGCTCTGGAGGTACTCA
LRRN4	CACACCCACCTTGCTTCAAC	CATGCAGCTATCGTGCGAGA
KCNG3	GAGTCACCTTGAGGGTACTTAGA	CATAACCATCTCTCGGTAGCAAC
FAM228B	GGCTGAAACTGCCTACAAGAT	TAGCACAGCGGTTCTCTTTCT
IGSF10	TTGGAGTTTGCCTGATGGAAC	CGCTACCCCAACTTTGTTGAAG
TGFBR3	GTGTTCCCTCCAAAGTGCAAC	AGCTCGATGATGTGTACTTCCT
CCDC170	GCTCAGTGCTGTAGAAGCAAA	TTGTGAGGTTGTGACTGCATC

mRNAs and lncRNAs reverse transcription and real-time quantitative PCR kits were purchased from Guangzhou RiboBio Biotechnology Co., Ltd.

Supplemental text 1: Information of LOC102724169

KeggID: hsa:102724169

Description: uncharacterized LOC102724169

>LOC102724169
TCTCCCATCTGGCCTTCTGACTTAGGAATCCGCTAACGTCTGCCCTCGCTTTCCCAAATA
ATCCATCGCTCCCACGTACTATTTGGTTTTCTGAGCCTGGTGCGGGGTCTGGCCTCGGGC
TGTGCAGCGTAAGTAGCTATGCAGGGCATTTATCCTGCAAGCTGCAGTGAGCCGAGACCC
TAAAGCTTGTTGCATCTGTTTGTCTTTGGAGGTAAAGGTGGTGTTATGTGTGTGTTTGCT
GGGGGGTGGTCTTTTGGAAATAGGGAGACTGGAGGAAAAATAAACAGGACATTGGTAAGT
GAAATAATATTGAATTAAAATTTTTCTGTTAAGGCAGTGAGCCCTGGTAAGAGATTTTTT
TTTTTTTCTTAATAAAAGCCCTTGTGTCTGCGCCTTACCAGCGAGACTTGATAAGGGTTA
CTTCCTCCAGCAAGAAGGTTAAAAGTAGCAGTGGAGCAGGAACTGCGGTATCAATACACG
GAAGATGGCAATAGGAGGAAAAAATACCTACTATTATACAAATAGCCGTGCTGTTTCCTT
GCTGAGAGAAAAACTGGCTATTGAAAAGCAGCGATTGAACTTGTGAACAATGGGGTGATT
TGAACTTGGAAAAGAAGGAAAAATGGATGCTTTTTCCTCTCACTAAATCCTCACAGCATT
GTGGACTCACACGAACCTTTAGAAATCATTATTCTTTCCATTTTTAATTGACAAATAAAA
GTTATATACATT
